# Annually modelling built-settlements between remotely-sensed observations using relative changes in subnational populations and lights at night

**DOI:** 10.1016/j.compenvurbsys.2019.101444

**Published:** 2020-03

**Authors:** Jeremiah J. Nieves, Alessandro Sorichetta, Catherine Linard, Maksym Bondarenko, Jessica E. Steele, Forrest R. Stevens, Andrea E. Gaughan, Alessandra Carioli, Donna J. Clarke, Thomas Esch, Andrew J. Tatem

**Affiliations:** a WorldPop Project, UK; b Department of Geography and Environment, University of Southampton, UK; c Department of Geography, Université de Namur, Belgium; d Department of Geography and Geosciences, University of Louisville, KY, USA; e German Aerospace Center (DLR), German Remote Sensing Data Center (DFD), Oberpfaffenhofen, Germany

**Keywords:** Built-settlements, Urban features, Spatial growth, Random forest, Dasymetric modelling, Population

## Abstract

Mapping urban features/human built-settlement extents at the annual time step has a wide variety of applications in demography, public health, sustainable development, and many other fields. Recently, while more multitemporal urban features/human built-settlement datasets have become available, issues still exist in remotely-sensed imagery due to spatial and temporal coverage, adverse atmospheric conditions, and expenses involved in producing such datasets. Remotely-sensed annual time-series of urban/built-settlement extents therefore do not yet exist and cover more than specific local areas or city-based regions. Moreover, while a few high-resolution global datasets of urban/built-settlement extents exist for key years, the observed date often deviates many years from the assigned one. These challenges make it difficult to increase temporal coverage while maintaining high fidelity in the spatial resolution. Here we describe an interpolative and flexible modelling framework for producing annual built-settlement extents. We use a combined technique of random forest and spatio-temporal dasymetric modelling with open source subnational data to produce annual 100 m × 100 m resolution binary built-settlement datasets in four test countries located in varying environmental and developmental contexts for test periods of five-year gaps. We find that in the majority of years, across all study areas, the model correctly identified between 85 and 99% of pixels that transition to built-settlement. Additionally, with few exceptions, the model substantially out performed a model that gave every pixel equal chance of transitioning to built-settlement in each year. This modelling framework shows strong promise for filling gaps in cross-sectional urban features/built-settlement datasets derived from remotely-sensed imagery, provides a base upon which to create urban future/built-settlement extent projections, and enables further exploration of the relationships between urban/built-settlement area and population dynamics.

## Highlights

• Model framework correctly predicts 85–95% of built-settlement transitions for the most years.
• Model framework shows consistent performance across variety of settings.
• Model framework can fill in temporal coverage gaps of remotely sensed urban feature datasets.
• Relative changes in population count and density are sufficient predictors of built-settlement expansion.

## 1 Introduction

Having time series of regular and consistent observations of built settlement extents is important given that forecasted growth of populations within dense urban areas are expected to continue through 2050, with much of that increase will occur within Africa and Asia (Angel, Sheppard, & Civco, 2005; United Nations, 2015b). Further, rapidly changing magnitudes and distributions of both built-settlements and populations have significant implications for sustainability (Cohen, 2006), climate change (McGranahan, Balk, & Anderson, 2007; Stephenson, Newman, & Mayhew, 2010), and public health (Chongsuvivatwong et al., 2011; Dhingra et al., 2016), amongst others. At local and regional levels, the availability (or non-availability) and accuracy of built-settlement extent data affect measured population distributions, densities, and classified landscape types (e.g. urban, peri-urban, and rural) used to inform and shape policies. The 2030 Agenda for Sustainable Development, which have a focus on accounting for and including “all people everywhere”, reinforced the need for readily and globally available baseline data to guide efforts and measure progress toward its Sustainable Development Goals (SDGs) (United Nations, 2016).

**Fig. 1:**
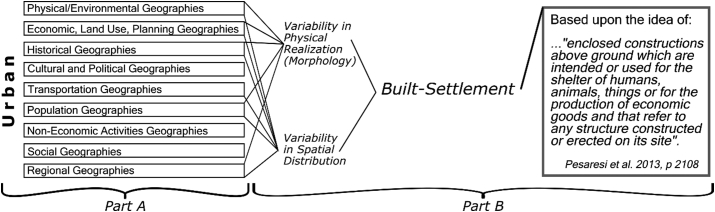
Generalized concept of “urban” (Part A), the conceptual relations and definition of “built-settlement” (Part B) as related to urban, and the broad, non-exhaustive contributing factors that make these concepts.

Urban has been defined in many ways across many fields with different definitions existing even within the same field depending upon the specific application. Many countries define urban as a function of some population magnitude/density threshold or based upon administrative jurisdictions and functional economic areas and activities (United Nations, 2015a, United Nations, 2018). While not conducive to applications requiring global consistency in definitions (Potere & Schneider, 2007), none of these definitions of the concept of urban are objectively wrong. Urban, whose formal yet vague language definition is “of, relating to, characteristic of, or constituting a city” (Merriam-Webster, 2019) is a complex amalgamation of the physical environment, population, economics, movements, and connectivity (Angel, Parent, Civco, Blei, & Potere, 2011; Berechman & Gordon, 1986; Burgess, 1925; Cohen, 2004, Cohen, 2006; De Haas, 2010; Dyson, 2011; Gottman, 1957; Harris & Ullman, 1945; Hoyt, 1939; Ledent, 1982; Meyer & Turner, 1992; Parr, 2004; Pozzi & Small, 2005; Schneider, Friedl, & Potere, 2010; Seto, Fragkias, Guneralp, & Reilly, 2011; Southworth, 1995; Von Thunen, 1966; Zelinsky, 1971). Fig. 1, Part A gives a generalized diagrammatic view of the factors contributing to the concept of urban.

As a result, many studies have turned to a definition based upon the remotely-sensed (RS) physical features of urban areas, i.e. the built-environment. However, even reducing the definitional scope of urban to its physical dimension, the form of built-environment can widely vary across space and time due to the types of materials used, differences in urban morphology, and the surrounding environmental context (Schneider et al., 2010; Schneider & Woodcock, 2008; Small, 2009). Initially, remotely sensed urban definitions were optically-based thematic classifications of land cover, typically capturing the “built-environment,” including buildings, roads, runways, and, sometimes erroneously, bare soil (Bartholomé & Belward, 2005; Potere, Schneider, Angel, & Civco, 2009; Schneider, Friedl, McIver, & Woodcock, 2003; Schneider et al., 2010). Other definitions have utilized urban delineated extents and densities based upon Lights-At-Night (LAN) data (Elvidge, Baugh, Kihn, Kroehl, & Davis, 1997; Henderson, Yeh, Gong, Elvidge, & Baugh, 2003; Liu, de Sherbinin, & Zhan, 2019; Shi et al., 2014; Small, Elvidge, Balk, & Montgomery, 2011; Small, Pozzi, & Elvidge, 2005; Wicht & Kuffer, 2019). Later improvements using supporting information about the surrounding environment and vegetation during post-processing helped better discern the true built-environment from the surrounding land covers (Schneider et al., 2010). Other notable advances include the use of high resolution orthographic imagery to detect subtle short-term built-environment change in China (Huang, Wen, Li, & Qin, 2017) and the use of Landsat imagery to create multi-temporal thematic representations of the built environment across the globe (Liu et al., 2018).

Coinciding with advances in imagery, statistical methods, and computational resource availability, high-resolution datasets with global extent have been created either through combining multi-source optical imagery with contrast detection methods (Pesaresi et al., 2016, Pesaresi et al., 2013) or utilizing Synthetic Aperture Radar (SAR) data with object-based image analysis to refine the capture of urban features, with a focus on vertical human-made structures (i.e. built-settlements), while attempting to exclude other anthropogenic land covers (Esch et al., 2013). However, it remains a challenge to produce consistent global urban feature/built-settlement products while maintaining high temporal and spatial fidelity, meaning most of the global multi-temporal urban feature/built-settlement data sets refer to few time points across a larger time period. Further, the cost of producing these data remains relatively high (Esch et al., 2018) and there can be pre-existing gaps in the input data, due to selected sensor/platform characteristics or problems and adverse atmospheric conditions, prior to the other fidelity considerations. While there is now a global abundance of high-resolution imagery, with various instruments and revisit times on various platforms, not all imagery are suitable for producing high-frequency global urban feature/built-settlement data sets. This is either because of the aforementioned reasons and/or because the processing cost may not be viable or the funds for such endeavours may not be available.

One way to address these issues is to leverage years where RS-based urban feature/built-settlement extractions with high spatial fidelity are available and interpolate for missing time points and areas of interest by modelling between available years. Overall, urban feature/built-settlement growth models have disproportionately focused on high-income countries, which have different dynamics than low- and middle-income countries (Angel et al., 2005; Linard, Tatem, & Gilbert, 2013; Seto et al., 2011; United Nations, 2015b), and most have been limited to city or regionally specific models (Barredo, Demicheli, Lavalle, Kasanko, & McCormick, 2004; Batty & Xie, 1994; Clarke & Gaydos, 1998; Clarke, Hoppen, & Gaydos, 1997; Huang, Hu, Li, & Wang, 2018; Leao, Bishop, & Evans, 2004; Linard et al., 2013; Sante, Garcia, Miranda, & Crecente, 2010; White and Engelen, 1997, White and Engelen, 2000). Previous methods of modelling urban feature/built-settlement growth across space and time at the continental and global scales include land cover/land use transition models (Tayyebi et al., 2013; Verburg, Schot, Dijst, & Veldkamp, 2004) and cellular automata models (Batty, 2009; Sante et al., 2010; Verburg et al., 2002), with features or thematic classes extracted from remotely-sensed imagery being the primary source of cross-sectional input for these models (Esch et al., 2013; Patel et al., 2015; Pesaresi et al., 2016, Pesaresi et al., 2013; Schneider et al., 2010). Readers are referred to Li and Gong (2016) and Sante et al. (2010) for comprehensive reviews of the wide field of cellular automata models as applied to urban feature/built-settlement growth modelling. Of the few models predicting urban feature/built-settlement growth across the globe within a standardized framework, almost none provided explicit spatial prediction finer than country level summaries (Angel et al., 2011; Seto et al., 2011). Global models that did provide explicit spatial predictions, did not allow local sub-national variations to drive the modelled changes or had not been assessed against comparable existing datasets (Angel et al., 2011; Goldewijk, Beusen, & Janssen, 2010; Linard et al., 2013; Seto, Guneralp, & Hutyra, 2012).

Building upon the previous work of these models, in this study, we leveraged the recently available multi-temporal global urban feature/built-settlement datasets, global environmental datasets, subnational census-based population data, and computational methods to develop a flexible globally applicable modelling framework based upon random forest classification trees, population growth curves, and cubic splines. Our specific objectives were to i) determine if random forests can reasonably predict the probability of non-BS to BS transition probabilities, ii) use the predicted surface of non-BS-to-BS transition probabilities as input to an automated framework to annually estimate spatially explicit BS extents using sub-nationally driven geospatial covariates and population counts, iii) validate the model performance and validate the model outputs.

Because the focus of this study is on modelling urban feature/built-settlement extents that better represent where people may be located, we adopted the Global Human Settlement Layer (GHSL) concept of “built-settlement” (BS) (Fig. 1), which is defined as, “…enclosed constructions above ground which are intended for the shelter of humans, animals, things or for the production of economic goods and that refer to any structure constructed or erected on its site.” (Pesaresi et al., 2013, p. 2013). We further generalized the definition of BS to include other datasets that attempt to represent buildings associated with human activities while attempting to exclude more general impervious surfaces, such as roads, parking lots, and runways. With the adopted definition of BS, the analogue to the process of “urbanization” is taken within a remote sensing context to be the physical transition from a non-BS area to a BS area.

## 2 Methods and data

### 2.1 Study areas

We selected four countries (Table 1) from across the globe to capture a variety of BS morphologies, contexts, and evolutions as well as to demonstrate the flexibility of the model for differing spatial detail of input census-based population data, as measured by the average spatial resolution (Tobler, Deichmann, Gottsegen, & Maloy, 1997). The countries selected here were Panama, Switzerland, Uganda, and Vietnam, which are located in rather contrasting geographies and environmental/urban biomes (Schneider et al., 2010) and represent quite different cultural and developmental contexts (from low-, middle-, to high-income countries). While it is known that many urban feature datasets have difficulty classifying the built-environment in arid regions, this is more a concern of the selected representation of BS input into the modelling framework rather than an issue for the framework itself; an inaccurate or “noisy” input will always produce poor results in an interpolative model.

**Table 1:** Summary of built-settlement transition data by country and period. Areal units here are pixels (~100 m) as that is the unit handled by the model which looks at relative areal changes as opposed to absolute areal changes.

Country	Average Spatial Resolution^a^	Period	Initial Non-Built Area (pixels)	Period Transition Prevalence
Panama	10.9 km	2000–2005	8,901,004	0.03%
		2005–2010	8,898,679	0.09%
		2010–2015	8,890,339	0.75%
Switzerland	3.9 km	2000–2005	6,816,510	1.56%
		2005–2010	6,710,069	0.08%
		2010–2015	6,704,973	0.01%
Uganda	12.2 km	2000–2005	28,231,555	0.07%
		2005–2010	28,210,425	0.04%
		2010–2015	28,200,084	0.04%
Vietnam	21.7 km	2000–2005	40,108,425	0.11%
		2005–2010	40,063,545	0.18%
		2010–2015	39,990,858	0.38%

a Average spatial resolution is the square root of the average subnational area, in km, and can be thought of as analogous to pixel resolution with smaller values indicating finer areal data and vice versa (Tobler et al., 1997).

### 2.2 Built-settlement data

We chose to use the “Urban areas” thematic class, class 190, from the ESA CCI land cover 300 m annual global land cover time-series from 1992 to 2015 dataset (https://www.esa-landcover-cci.org/; hereafter ESA) for our study. It was selected for its annual coverage, allowing for the withholding of years in the model training process for validation of latter modelled outputs. For our period of interest, 2000 to 2015, the ESA time-series includes annual 10 arc sec resolution (~300 m at Equator) datasets produced by looking for thematic class changes from a baseline land cover map, obtained using MERIS imagery, using 30 arc sec (~1 km at the Equator) SPOT VGT imagery (1999–2013) and PROBA-V imagery (2014–2015) (UCL Geomatics, 2017). Prior to 2004, detected changes are delineated at 30 arc sec resolution. Starting in 2004, if there are changes detected, then the individual pixels of change detected at 30 arc sec are further delineated using 10 arc sec MERIS or PROBA-V imagery (UCL Geomatics, 2017). To reduce false detections, changes must be observed over two years or more (UCL Geomatics, 2017). Furthermore, the GHSL (Pesaresi et al., 2016, Pesaresi et al., 2013) and Global Urban Footprint (GUF) (Esch et al., 2013) datasets are utilized in defining the extents of the ESA “Urban areas” class (UCL Geomatics, 2017), which thus incorporate elements of two BS datasets within the larger built-environment context. While still undergoing full validation, initial validation efforts estimate the 2015 “Urban areas” class user and producer accuracies between 86 and 88% and 51–60%, respectively (UCL Geomatics, 2017). We also tested and validated a single year, 2010 as predicted from the years 2000 and 2015, from an alpha version of the forthcoming multi-temporal World Settlement Footprint (WSF) dataset, known as WSF Evolution (Esch et al., 2018), and present the results in the Supplementary Material.

### 2.3 Population data

Annual population counts from 2000 to 2015 for subnational areas were provided by the Center for International Earth Science Information Network (CIESIN) in tabular format with unique IDs corresponding to unique subnational unit IDs (Doxsey-Whitfield et al., 2015). Populations and areas of the subnational units are based upon the Gridded Population of the World, version 4 (GPWv4) and as such follow the methods detailed in Doxsey-Whitfield et al. (2015) for the interpolation and extrapolation of population between 2000 and 2015, inclusive, using years of official counts or estimates.

### 2.4 Geospatial data

We selected a number of covariates based upon previous urban feature/built-environment models (Linard et al., 2013; Verburg et al., 2002; Verburg, de Koning, Kok, Veldkamp, & Bouma, 1999) to give the model information on the immediate environmental/land cover context and connectivity of urban feature/built-settlements. Ultimately, the model is not dependent on any specific geospatial covariates, retaining a level of flexibility for use in a wide variety of applications. For example, a minimal set of globally available predictive covariates to produces inputs for other modelling efforts while avoiding potential issues relating to endogeneity. In the case presented here, annually available covariates, or single time point covariates reasonably assumed to be time invariant, were used either in the direct calculation of transition probabilities or in the remainder of the disaggregative process (Table 2). As detailed in Lloyd et al. (2019), all covariates were pre-processed, appropriately resampled, and matched to a common spatial grid having a resolution of 3 arc sec; with the latter chosen as a compromise between the higher resolutions of some of the covariates (Table 2) and the ESA datasets. All data used to restrict the area of modelling and inform the redistribution of transitions are also detailed in Table 2. Further details on pre-processing of specific covariates are provided in the Appendices.

**Table 2:** Data used for estimating the annual number of non-BS to BS transitions at the unit level (i.e. demand quantification), predicting the pixel level probability surface of those transitions, and performing the spatial allocation procedures of the model.

Covariate	Variable Name (s) in Random Forest	Description	Use^b^, ^d^	Time Point (s)	Original Spatial Resolution (s)	Data Source (s)
Built-settlement^c^	esa_cls190	Binary BS extents	Demand Quantification and Spatial Allocation	2000 2005 2010 2015	10 arc sec	(ESA CCI, 2017)
DTE Built-settlement	esa_cls190_dst_*<year>*	Distance to the nearest BS edge	Spatial Allocation^d^	2000	10 arc sec	(ESA CCI, 2017)
Proportion Built-settlement 1,5,10,15	esa_cls190_prp_<*radius*>_*<year>*	Proportion of pixels that are BS within 1,5,10, or 15 pixel radius	Spatial Allocation^d^	2000	10 arc sec	(ESA CCI, 2017)
Elevation	Topo	Elevation of terrain	Spatial Allocation^d^	2000 – Time Invariant	3 arc sec	(Lehner, Verdin, & Jarvis, 2008)
Slope	Slope	Slope of terrain	Spatial Allocation^d^	2000 – Time Invariant	3 arc sec	(Lehner et al., 2008)
DTE Protected Areas Category 1	wdpa_cat1_dst_2015	Distance to the nearest level 1 protected area edge	Spatial Allocation^d^	2015	Vector	(U.N. Enviroment Programme World Conservation Monitoring Centre & IUCN World Commission on Protected Areas, 2015)
Water	–	Areas of water to restrict areas of model prediction	Restrictive Mask		5 arc sec	(Lamarche et al., 2017)
Subnational Population	–	Annual population by sub-national units	Demand Quantification	2000–2020, annually	Vector	(Doxsey-Whitfield et al., 2015)
Weighted Lights-at-Night (LAN)	–	Annual lagged and sub-national unit normalized LAN	Spatial Allocation	2000–2016, annually	30 arc sec (2000−2011) 15 arc sec (2012–2016)	DMSP (WorldPop, Department of Geography and Geosciences, Département de Géographie, & Center for International Earth Science Information Network (CIESIN), 2018; Zhang, Pandey, & Seto, 2016) VIIRS (Earth Observation Group NOAA National Geophysical Data Center, 2016; WorldPop et al., 2018)
Travel Time 50 k	tt50k	Travel time to the nearest city centre containing at least 50,000 people	Spatial Allocation^d^	2000	30 arc sec	(Nelson, 2008)
Urban Accessibility 2015	urbanaccessibility_2015	Travel time to the nearest city edge	Spatial Allocation^d^	2015	30 arc sec	(Weiss et al., 2018)
ESA CCI Land Cover (LC) Class^a^	ccilc_dst<*class number*>_<*year*>	Distance to nearest edge of individual land cover classes	Spatial Allocation^d^	2000	10 arc sec	(ESA CCI, 2017)
Distance to OpenStreet Map (OSM) Rivers	osmriv_dst	Distance to nearest OSM river feature	Spatial Allocation^d^	2017	Vector	(OpenStreetMap Contributers, 2017)
Distance to OpenStreet Map (OSM) Roads	osmroa_dst	Distance to nearest OSM road feature	Spatial Allocation^d^	2017	Vector	(OpenStreetMap Contributers, 2017)
Average Precipitation	wclin_prec	Mean Precipitation	Spatial Allocation^d^	1950–2000	30 arc sec	(Hijmans, Cameron, Parra, Jones, & Jarvis, 2005)
Average Temperature	wclim_temp	Mean temperature	Spatial Allocation^d^	1950–2000	30 arc sec	(Hijmans et al., 2005)

a Some classes were collapsed: 10–30 → 11; 40–120 → 40; 150–153 → 150; 160–180 → 160 (Sorichetta et al., 2015).

b Covariates involved in Demand Quantification were used to determine the demand for non-BS to BS transitions at the subnational unit level for every given year. Covariates involved in Spatial Allocation were either used as predictive covariates in the random forest calculated probabilities of transition (see d) or as a post-random forest year specific weight on those probabilities and the spatial allocation of transitions within each given unit area. Covariates used as restrictive masks prevented transitions from being allocated to these areas.

c In the dasymetric modelling process, the 2000, 2005, 2010, and 2015 binary BS data were utilized as observed points, but only derived covariates for 2000 were utilized in the random forest as predictive covariates.

d Used as predictive covariates in the random forest calculated probabilities of transition.

### 2.5 Built-settlement growth model (BSGM)

#### 2.5.1 Overview

Here we interpolated BS extents for every year between a set of RS-based observed years, *T* = {*t0, t*_*1*_*, t*_*2*_*, …, t1*} where *t0* is the initial RS-based observed year, *t1* is the final RS-based observed year, and all other times *t*_*k*_ are years lying between *t0* and *t1* for which we had RS-based observed BS extents. The time between any two RS-based observed time points *t* is referred to as a period, *p*, with all periods being a subset of *P*. Within this study, *T* = {2000, 2005, 2010, 2015} and *P* *=* {2000–2005, 2005–2010, 2010–2015} and, therefore, we are modelling across three periods, estimating BS extents for 12 years, based upon the input of four RS-based observed years. However, the interpolative BSGM modelling framework can handle any regularly spaced intra-period time-step if the input data corresponds.

**Fig. 2:**
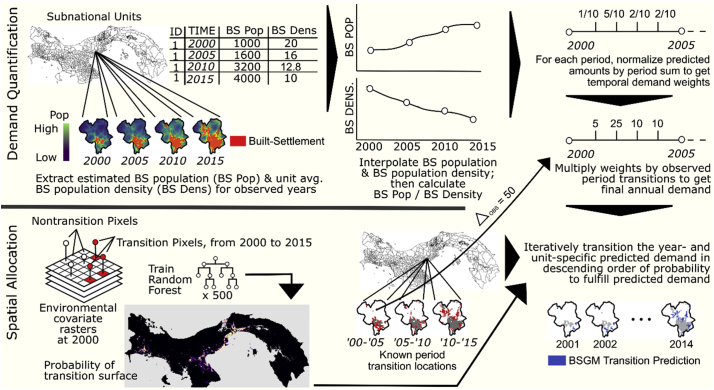
High-level example overview of the BSGM modelling framework process for interpolation using four RS-based observed years (2000, 2005, 2010, 2015) and predicting for all unobserved years in between. Note, example maps and numbers are not to scale.

The interpolative BSGM modelling framework has two main components: a demand quantification component and a spatial allocation component, as shown in Fig. 2.

We generalize the process to determine the number of non-BS to BS transitions for each year we are interpolating, i.e. demand quantification, independently for each subnational unit, hereafter unit, as follows:

1. Create a population map for all years in *T* (2000, 2005, 2010, 2015)*.*
2. At all RS-based observed years *t* in *T*, for each unit, extract the time- and unit-specific population count within the corresponding BS extents and derive the corresponding unit-average BS population density, i.e. lacking more precise information all BS pixels in a unit have the same BS population density (Fig. 2).
3. On a unit-by-unit basis, interpolate the extracted BS population count and BS population density for all years, *t*_*k*_, between each RS-based observed year *t* in *T* (Fig. 2).
4. Estimate year- and unit-specific number of expected non-BS-to-BS transitions based upon the corresponding predicted BS population and BS population density (Fig. 2).
5. Within each unit, for each period, create annual demand weights by normalizing the annual number of expected transitions (from step 4) by the sum of the period's annual number of expected transitions (Fig. 2).
6. For each unit and period, use the annual weights (from step five) to dasymetrically redistribute the period's total observed transitions to each year within the given period (Fig. 2). Repeat for all periods.

To spatially allocate, i.e. disaggregate, the estimated annual transitions, (from step 5) we first train a Random Forest (RF) model (Breiman, 2001) to produce a continuous surface representing the probability of a given pixel transitioning from non-BS to BS between *t0* and *t1*, i.e. 2000 and 2015 (Fig. 2). For every year, and independently for each unit, we utilized unit-normalized annually lagged lights-at-night (LAN) data to adjust the base RF-derived transition probabilities annually. Given that the BSGM modelling framework is interpolative, we limited the spatial allocation component to predicting transition probabilities in pixels that, based upon the input data, were observed to have transitioned within the given period (Fig. 2). For example, only pixels seen to have transitioned between 2000 and 2005 could be predicted as transitioning in 2001, 2002, 2003, or 2004. With this in mind, within each unit, we selected pixels with the *n*^th^ highest probabilities for transition, where *n* was equal to the number of pixels estimated to transition in that unit for that year. We then converted those pixels to BS, recorded the new BS extents, and used those extents as the basis for the next time-step of transitions. This resulted in a series of annual binary BS extent datasets. All modelling and analyses were carried out using R 3.4.2 (R Core Team, 2016) on the IRIDIS 4 high-performance computing cluster (see Appendices for the full process diagram and the Supplemental Materials for the modelling code).

#### 2.5.2 Demand quantification

First, we created population distribution datasets for all years *t* in T by using available time-specific covariates (see Appendices) and the method, described in Gaughan et al. (2016) and Stevens, Gaughan, Linard, and Tatem (2015), to dasymetrically redistribute the time-specific unit-based population counts to 3 arc sec grid pixels (Mennis, 2003; Mennis & Hultgren, 2006). Second, for each unit and year *t* in *T*, we extracted and summed the population counts spatially coincident with the BS extents, i.e. BS population counts, and derived the corresponding BS population density for use in the later stages of the demand quantification component. Third, for each year *t*_*p*_ within a given period *p*, we interpolated the BS population count of each unit *i*, i.e. *BSPOP*_*i*_*(t*_*p*_*)*, using logistic growth curves with year-specific total population, *K*_*i*_*(t*_*p*_*)*, as the dynamic carrying capacity (Booth, 2006; Meyer & Ausubel, 1999). See Appendices for rationale regarding the use of a logistic growth curve with a dynamic limiting factor. These curves were fit in a piecewise manner, i.e. one curve for each period *p* ∈ *P*. This is written in Eq. (1) as:

(1) $$\mathrm{BSPO}P_{i}\left(t_{p}\right)=K_{i}\left(t_{p}\right)*\frac{e^{r_{i}*t_{p}+C_{i}}}{1+e^{r_{i}*t_{p}+C_{i}}}$$

where *r*_*i*_ and *C*_*i*_ are determined by fitting a least-squares linear regression to the set of observed values corresponding the given period after having been transformed via Eq. (2):

(2) $$\ln\left(\frac{\mathrm{BSPO}P_{{\mathrm{it}}_{\mathrm{observed}}}}{K_{{\mathrm{it}}_{\mathrm{observed}}}-\mathrm{BSPO}P_{it_{\mathrm{observed}}}}\right)=r_{i}\left(t_{p}\right)+C_{i}$$

Fourth, to interpolate the unit-average BS population density for each unobserved year *t*_*k*_ between the years *t* in *T*, we fit natural cubic splines (McNeil, Trussell, & Turner, 1977) for each unit *i* across all unobserved years using the years *t* in *T* as the knots. Our priority being that the fit curve would match our values of observation, adapting to the data, i.e. non-parametric smoothing, rather than adapting the data to a specific distribution, i.e. parametric approach. See Appendices for more rationale on the use of cubic splines.

Finally, to begin estimating number of transitions, in each unobserved year *t*_*k*_ and for each unit *i*, we simply related the corresponding interpolated BS population and BS population density in Eq. (3):

(3) $$\hat{\mathrm{BSCN}T_{i}\left(t\right)}=\frac{\mathrm{BSPO}P_{i}\left(t\right)}{\mathrm{BS}D_{i}\left(t\right)}$$

where *BSD*_*i*_*(t)* is the unit-average BS population density at time *t*. See Appendices for how predicted “negative growth” resulting from Eqs. (1), (2), (3) was handled.

In order to maintain agreement with the input data, i.e. the RS-based observed BS extents, the sum of our annual estimated transitions needed to match the total number of observed transitions within a given period *p*. So, we reweighted the estimated transitions of each year on a unit-by-unit basis using the sum of the estimated transitions in the period *p*. To calculate the unit-and year-specific weight, *w*_*ip*_(*t*_*p*_), within the period *p*, we write the calculation in Eq. (4) as:

(4) $$w_{\mathrm{ip}}\left(t_{p}\right)=\frac{\hat{\mathrm{BSCN}T_{i}}\left(t_{p}\right)}{\sum_{1}^{k}\hat{\mathrm{BSCN}T_{i}}\left(t_{p}\right)}$$

where *t*_*p*_ is again relative to the given period *p*, from 1 to the last year *k*, and all *w*_*ip*_ for a given unit *i* and period *p* sum to one.

Then, using these weights, we carried out a temporal dasymetric redistribution of the total observed transitions from the larger source period *p*, e.g. 2000–2005, to the individual unobserved years, e.g. 2001, …, 2004. To obtain the final temporally disaggregated transitions, *BSCNT*_*iFINAL*_(*t*_*p*_), we multiplied the unit- and year-specific weight, *w*_*ip*_(*t*_*p*_), by the corresponding period *p*'s observed transitions, *∆BSCNT*_*ip*_, rounding to the nearest whole number for each year, as shown in Eq. (5) (see Appendices for obtaining agreement with rounding differences).

(5) $$\hat{\mathrm{BSCN}{T_{i}}_{\mathrm{FINAL}}\left(t_{p}\right)}=\mathrm{round}\left(w_{\mathrm{ip}}\left(t_{p}\right)*∆\mathrm{BSCN}T_{\mathrm{ip}}\right)$$

#### 2.5.3 Spatial allocation

We utilized a RF model to accurately and efficiently model, across each country, the probability of each pixel transitioning from non-BS-to-BS. Importance of individual covariates in a classification random forest are typically measured by the average decrease in the Gini impurity, the probability of incorrectly classifying a random selected element of the dataset if it were randomly assigned label based upon the distribution of classes in the dataset (Breiman, 2001). A RF model was selected for its robustness to noise, its automatability and efficiency, and its ability to capture non-linear and complex interactions c. Furthermore, Kamusoko and Gamba (2015) showed that RFs have been shown to perform equally to, if not better, than other methods, (including support vector machines and logistic regression) used for predicting the probability of transitioning from non-built-environment to built-environment.

The binary dataset of non-BS-to-BS transition constitutes an intrinsic “imbalanced set” (He & Garcia, 2009), i.e. there are many more non-transitions than transitions. So, we adopted a stratified random over/under-sampling method (He & Garcia, 2009), similar to (Linard et al., 2013), as follows: (i) randomly sample 80% of the pixels observed to have transitioned between 2000 and 2015, up to 50,000 and, (ii) randomly sample an equal number of pixels that have not transitioned during the same time span. We then used these training sets and spatially and temporally coincident covariates to train a RF model for each country and predicted the corresponding surface of non-BS-to-BS transition probabilities. All covariates used were retained in the final model. These probabilities have a value between 0 and 1 and represent the posterior probability of a pixel being classified by the RF model as transitioning between *t0*, 2000, and *t1*, 2015 *c*.

We then refined these probabilities to annual probabilities using annual ancillary information. Given that changes in LAN brightness have been found to be good indicators of population and urban growth (Zhang & Seto, 2011), we adjusted the RF-derived transition probabilities using annual weights based upon unit-normalized annual average LAN brightness differences prior to spatially disaggregating the estimated annual non-BS-to-BS transitions from the demand quantification component. The rationale being that larger increases in average annual brightness for a given pixel, relative to all other pixels within the same unit, represent a higher relative probability of non-BS-to-BS transitions for that pixel and vice versa.

To create these annual spatial weights, we first calculated the annual lags of the LAN radiance values and rescaled the differences between 0 (unit's lowest value) and 1 (unit's highest value). This rescaling was based upon the values of all pixels *M* within a given unit *i* for a given lag *l*, where the number of lags is equal to the number of years minus one, e.g. for 2000 to 2015 we have 14 lags beginning with 2001 minus 2000. This calculation for a given pixel *m*, where *m*∈*M* pixels total in the unit, can be written as:

(6) $$\mathrm{wLA}N_{i,m,l}=\frac{\mathrm{la}g_{i,m,l}-\min\left(\mathrm{la}g_{i,M,l}\right)}{\max\left(\mathrm{la}g_{i,M,l}\right)-\min\left(\mathrm{la}g_{i,M,l}\right)}$$

where *lag*_*m*, *l*_ = *LAN*_*m*, *τ*_ − *LAN*_*m*, *τ*−1_ and *τ* represents the most recent year of the lag *l*, e.g. for lag 2001–2000 *τ* would be 2001. We then calculated year specific transition probabilities for every pixel known to have transitioned, *j*, using Eq. (7):

(7) $$P_{\mathrm{adj}}{\left(\mathrm{transition}\right)}_{\mathrm{ijt}}=\mathrm{wLA}N_{\mathrm{ijt}}*P{\left(\mathrm{transition}\right)}_{\mathrm{ij}}$$

where *P*(*transition*)_*ij*_ is the RF-derived transition probability for observed transition pixel *j* in unit *i* and *P*_*adj*_(*transition*)_*ijt*_ is the corresponding resultant adjusted transition probability for year *t*:

Using these adjusted probabilities, we then spatially disaggregated the estimated annual transitions, from the demand quantification component, within each unit. Given that the non-BS-to-BS transition process is iterative in nature, we began by taking the extents of the previous year. Within each unit *i* and for each period *p*, we limited the location(s) where transitions could be allocated to pixels *j* as defined by the RS-based observed BS extents. For all pixels *j*, assuming they were not transitioned in previously iterated years, we retrieved the adjusted transition probabilities and, similar to previous models (Linard et al., 2013; Tayyebi et al., 2013), we assumed pixels with a higher probability of transition were more likely to transition before pixels with lower probabilities. We selected the *n*^th^ highest probabilities from the pixels *J* in unit *i*, where *n* was equal to $\hat{\mathrm{BSCN}{T_{i}}_{\mathrm{FINAL}}}$, changed the value of those *n* pixels to represent a non-BS-to-BS transition, and output the union of the new transitions and previous BS extents as the predicted BS extents for that year. We repeated this procedure using the newly produced extents for the preceding year as the base BS extent for the next year's transition procedure, until all years for the given period *p* were processed and then the entire procedure was repeated until all periods *p* in *P* had been processed, resulting in annual modelled BS extents.

### 2.6 Analyses

#### 2.6.1 Validation and comparison metrics

While the RF produces its own validation estimates (Breiman, 2001), we tested the accuracy of the RF classifier by randomly sampling 100,000 pixels, not utilized in the training of the RF, for validation. We selected this sample size as we were able to obtain sample prevalence rates equal to the known true prevalence rates of each country while still maintaining efficiency. Based on this sample, we plotted Receiver Operator Curves (ROCs) and, given the imbalanced data (He & Garcia, 2009; Saito & Rehmsmeier, 2015)**,** Precision Recall Curves (PRCs) with simulated perfect and random classifier curves for comparison.

Here we validated the modelled BS extents to all withheld ESA RS-based BS extents corresponding to the unobserved years between 2000 and 2015, i.e. 2001–2004, 2006–2009, and 2011–2014. Here “True” represents agreement of the BSGM-based BS extents to the temporally corresponding withheld annual ESA RS-based BS extents and vice versa. For every year of prediction, we determined whether a pixel was True Positive (TP), False Positive (FP), False Negative (FN), or True Negative (TN). Pixels used for validation of the modelled BS extents were limited only to pixels observed transitioning from non-BS to BS between the modelled periods for two related reasons:

1) Being an interpolative model, we constrained the areas of possible transition to only the areas of observed transition. This limited the spatial uncertainty of the model between 2000 and 2005, 2005 to 2010, and 2010 to 2015 to no worse than the input data, although temporal uncertainty for any specific year between those periods remained.
2) Given that we masked our predictions to only pixels we knew transitioned, if we were to have included pixels that we knew not to have transitioned, we would have grossly and erroneously inflated the error metrics.

We calculated contingency table-based metrics to evaluate classification agreement based primarily on the F_1_ score (Table 3) which is the harmonic mean of recall and precision, the quantity disagreement (R.G. Pontius & Millones, 2011), and the allocation disagreement (R.G. Pontius & Millones, 2011). We aggregated the pixel level results (See Supplemental Materials), to the unit level and calculated the same metrics since precision, and by extension F_1_, is sensitive to the corresponding prevalence and is subject to the Modifiable Areal Unit Problem (MAUP) (Openshaw, 1984).The MAUP not only reduces variance in value distributions the more the data are aggregated from their original resolution (Openshaw, 1984), but will result in different prevalences within different units, i.e. zonal, configurations. The equations of the metrics calculated are listed in Table 3.

**Table 3:** Classification agreement metrics. The F1-score is interpreted as the harmonic mean of precision and recall. TP is “True Positive”, FP is “False Positive”, FN is “False Negative”, and TN is “True Negative.”

Metric	Equation	Range and Interpretation
Recall (Sensitivity) (Rogan & Gladen, 1978)	$\frac{\mathrm{TP}}{\mathrm{TP}+\mathrm{FN}}$	0 (no recall) – 1 (perfect recall)
Specificity (Rogan & Gladen, 1978)	$\frac{\mathrm{TN}}{\mathrm{FP}+\mathrm{TN}}$	0 (no specificity) – 1 (perfect specificity)
Quantity Disagreement (R.G. Pontius & Millones, 2011)	$\frac{\left|\frac{\mathrm{FN}-\mathrm{FP}}{\mathrm{TP}+\mathrm{FP}+\mathrm{FN}+\mathrm{TN}}\right|+\left|\frac{\mathrm{FP}-\mathrm{FN}}{\mathrm{TP}+\mathrm{FP}+\mathrm{FN}+\mathrm{TN}}\right|}{2}$	0 (no disagreement) – 1 (complete disagreement)
Allocation Disagreement (R.G. Pontius & Millones, 2011)	$2*\min\left(\frac{\mathrm{FP}}{\mathrm{TP}+\mathrm{FP}+\mathrm{FN}+\mathrm{TN}},\frac{\mathrm{FN}}{\mathrm{TP}+\mathrm{FP}+\mathrm{FN}+\mathrm{TN}}\right)$	0 (no disagreement) – 1 (complete disagreement)
F_1_ score	$\frac{2*\frac{\mathrm{TP}}{\mathrm{TP}+\mathrm{FP}}*\frac{\mathrm{TP}}{\mathrm{TP}+\mathrm{FN}}}{\frac{\mathrm{TP}}{\mathrm{TP}+\mathrm{FP}}+\frac{\mathrm{TP}}{\mathrm{TP}+\mathrm{FN}}}$	0 (worst) – 1 (best)

As suggested by Pontius, Shusas, and McEachern (2004), to assess the predictive ability of the BSGM modelling framework, we compared it to a naive (basic) model that randomly assigns the transitions to a year within the given period, with every year having an equal likelihood, and carried out predictions for each year within pixels that were known to have transitioned for comparability with our framework. Again, we determined whether each pixel was a TP, FP, FN, or TN and calculated metrics to compare the BSGM-based BS extents and the BS extents produced using the naive model for each country at the pixel level, and at the unit level. The naive model was bootstrapped 500 times based upon resource limits and prediction stability, for each year and was specific to each country.

## 3 Results

Across all study areas, two-thirds of the modelled years correctly predicted between 85 and 99% of transition pixels. For all years, again at the pixel level, the BSGM-based BS extents displayed low quantity and allocation disagreement in both absolute and relative terms. Similarly, the pixel level F1 score, with few exceptions, was higher than the one calculated for the BS extents produced using the naive model, but had more variance in absolute terms of performance. Comparable results between were found at the unit level (See Appendices), with relatively higher performance in the middle and later years of the study period.

### 3.1 RF performance

**Fig. 3:**
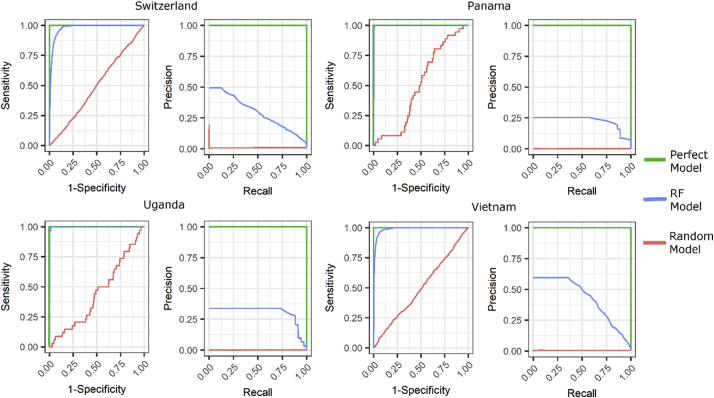
Receiver Operator Curve (left plots) and Precision Recall Curves (right plots) with the RF model performance, blue lines, against a random model (red lines), and a perfect model (green lines), for each modelled country. (For interpretation of the references to colour in this figure legend, the reader is referred to the web version of this article.)

The ROC plots (left plots in Fig. 3) show that the RFs approach the performance of the theoretical perfect model. However, given the imbalanced data, the PRC plots (right plots in Fig. 3) show a more nuanced picture of performance where a maximum level of precision is quickly achieved, remains steady up to a certain value of recall that varies by study area, and then quickly decreases with increasing recall.

**Fig. 4:**
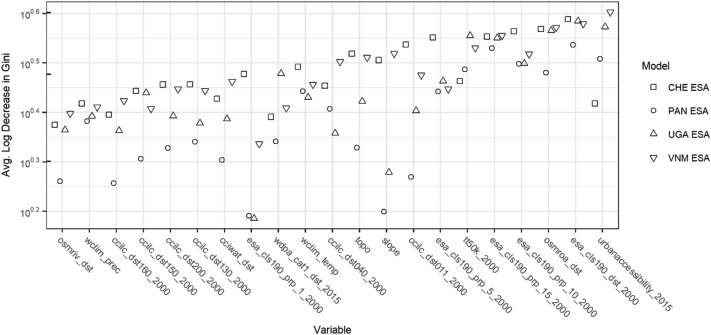
Random forest covariate importance as measured by the average log decrease in the Gini impurity when the covariate is used as the splitting criteria at nodes, for Swizerland (CHE) ESA, Panama (PAN) ESA, Uganda (UGA) ESA, and Vietnam (VNM). Higher values indicate better predictive performance of covariate. Refer to Table 2 for covariate names.

Of the covariates informing the RF models, we consistently saw that the most important predictors of a pixel transitioning from non-BS to BS (Fig. 4) were covariates related to distance (“esa_cls190_dst_2000”) and local density of BS (“esa_cls190_prp_5_2000”, “esa_cls190_prp_10_2000”, and “esa_cls190_prp_15_2000”) established at the beginning of the overall study period, i.e. 2000. Other important predictors included connectivity of BS extents (“tt50k_2000”) at the beginning or approximately end (“urbanaccessibility_2015” and “osmroa_dst”) of the study period (Fig. 4).

### 3.2 Predicted BS extents results

Examining the proportion of pixels known to transition that were predicted correctly (Table 4), we show that out of 48 modelled BS extents (corresponding to 12 years across four countries), 39 of those had correctly predicted proportions between 0.80 and 0.99 (green) with 25 of them having proportions over 0.90. Modelled extents ranged from 0.57 to 0.99 of pixels predicted correctly (Table 4). Note that one minus the proportion correct is equal to the total disagreement of the predicted pixels, i.e. the sum of the quantity and allocation disagreement (R.G. Pontius & Millones, 2011).

**Table 4:**
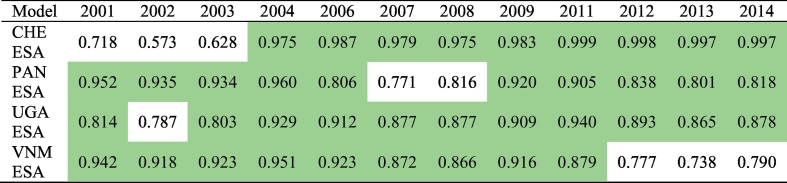
Proportion of transition pixels predicted correctly by the BSGM modelling framework by year for Switzerland (CHE, Panama (PAN), Uganda (UGA), and Vietnam (VNM). Modelled extents with proportions greater than or equal to 0.80 are highlighted in green.

**Fig. 5:**
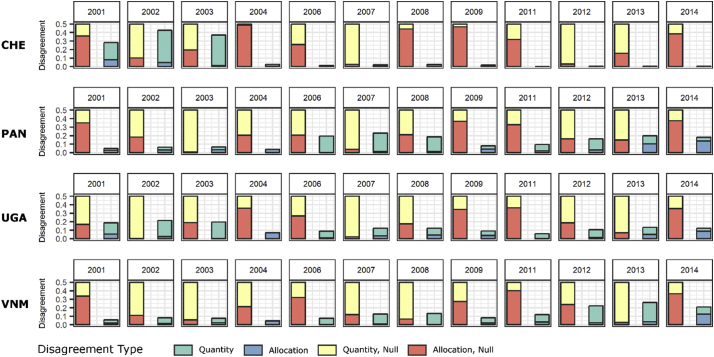
Pixel-level quantity and allocation disagreement of BSGM and naive models for Switzerland (CHE), Panama (PAN), Uganda (UGA), and Vietnam (VNM) as compared to a naive model, given in yellow and red. Full annual contingency data and metrics in supplemental materials. (For interpretation of the references to colour in this figure legend, the reader is referred to the web version of this article.)

Further examining source of disagreement, we display the quantity and allocation disagreement between the BSGM-based RS extents and validation set, i.e. ESA RS-based BS extents, as well as the corresponding disagreements with the BS extents produced using the naïve model (Fig. 5). We show that for all modelled years the total disagreement is substantially less than that of the naive model and the disagreement produced by the BSGM modelling framework is predominantly due to quantity error (Fig. 5). However, there does appear to be a pattern of increasing disagreement due to allocation error after 2010. Identical analyses for the early WSF Evolution data are provided in Appendices.

**Fig. 6:**
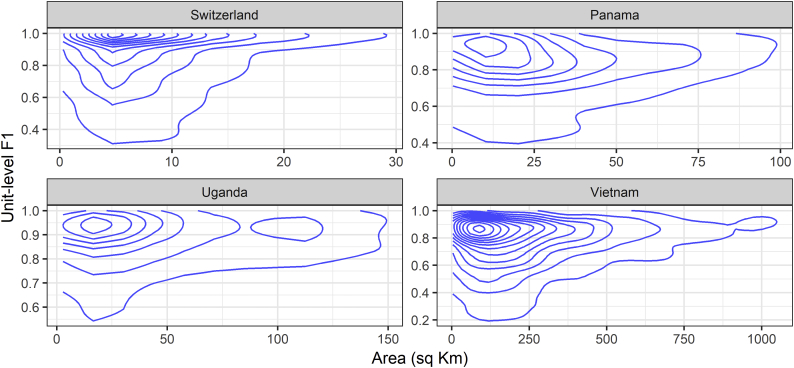
Contour density plot of unit-level F1 scores by country across all predicted years. Created using a two-dimensional kernel density estimation (Venables & Ripley, 2002).

While our ESA RS-based BS extents data does not give information on the true size of settlements on the ground, we can leverage the fact that our subnational units are derived from census boundaries (Doxsey-Whitfield et al., 2015), which are known to typically be smaller in areas of larger settlements and larger in areas of more fragmented smaller settlements, to begin understanding how the framework is operating across the continuum of settlement size. Looking at the contour density plots of F1 unit-level scores across all years for each country plotted against the corresponding subnational unit area, in Fig. 6, we can see that higher scores are clustered for units with smaller areas across each country, although, with the exception of Uganda, the framework shows good density and performance over a range of unit sizes. Less variance in performance for larger units is likely due to the smaller amount of transitions seen in these units, decreasing the probabilities for error by the interpolative BSGM modelling framework.

**Fig. 7:**
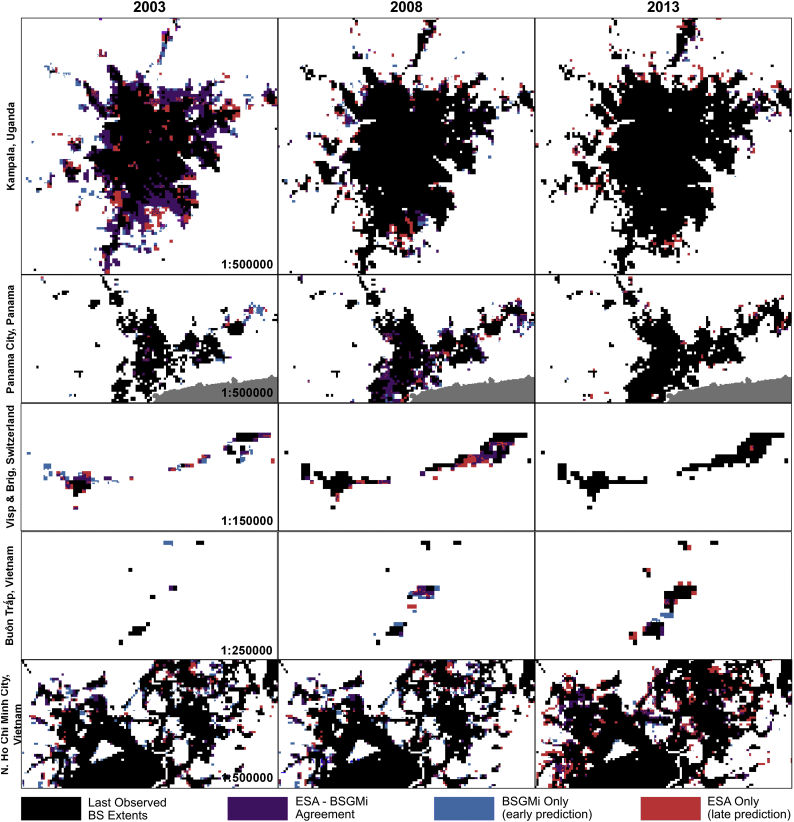
Selected BSGM-based BS extent and ESA RS-based BS-extent used for validation across the four countries for the approximate mid-point years of each period – 2003, 2008, 2013. “ESA Only” represents BS pixels in the validation dataset not classified as BS pixels in the corresponding BSGM-based BS extent. “BSGMi Only” represents BS pixels in the BSGM-based BS extent not classified as BS pixels in the validation dataset.

Examining examples of the annual BSGM-based BS extents against the corresponding annual ESA RS-based BS extents for the mid-point of each period, which should theoretically be the worst simply by being the furthest year from any observation we note a few things of interest. First, there are relatively large amounts of agreement whether for small or large settlements (with Visp being a town of less than 10,000). Second, the framework seems to predict “infill” growth, e.g. Kampala in 2003 and North Ho Chi Minh City in 2013, later than indicated by the corresponding ESA RS-based extents (Fig. 7, in red). Lastly, it appears that the BSGM modelling framework is temporally conservative in that it is not predicting relatively large amounts of pixels too early (Fig. 7, in blue). Of course, the model performance can vary from unit-to-unit and year-to-year, and we provide the entire annual BSGM-based BS extents in GeoTiff format in the Supplemental Materials:

## 4 Discussion

Here we have shown that the BSGM framework is capable of filling gaps in time-series of built-settlement datasets by estimating the extents in between RS-based imagery using relative changes in BS population and BS population density combined with environmental covariates. The BSGM modelling framework approximates patterns of BS growth through time with good agreement to its input BS extent dataset for most years, both at the pixel and unit level (Table 4, Fig. 5, Fig. 6, Fig. 7, and Appendices). This emphasizes the strength of incorporating the use of an interpolative model, such as the BSGM modelling framework, as opposed to solely using urban feature datasets that are largely imagery-dependent. While still the gold standard, these imagery-based datasets may be affected by adverse atmospheric conditions, limited sensor revisits, or the need for more resource intensive imagery-based interpolation or extraction methods. This framework, and resultant output data, can be used for better modelling population distribution through time, inform future extractions of BS from imagery, help facilitate intervention/planning/monitoring of development goals, and potentially serve as a platform for simulating different transition paths through time and investigating correlates of BS spatial growth.

However, this validation design has limits. The agreements and disagreements here are generated by how well the BSGM model replicates the spatio-temporal data patterns of the input ESA BS extents and does not state anything about the accuracy of the BSGM-predicted extents as compared to ground truth. Even if we possessed accurate and time-specific BS ground truth extents with complete spatial coverage, given that the BSGM is an interpolative modelling framework it would be difficult to determine if any error originated from the model or was propagated from the input BS extents. Performance as assessed by ground truth would be highly sensitive to the chosen BS extents input to the BSGM. We assume if the BSGM can accurately replicate and interpolate the data patterns of the input dataset, then the end user can have some confidence that validation metrics provided by the original data producers, e.g. ESA, are likely to hold. However, ground truth accuracy is important for some end users, and we encourage them to assess the BSGM output accordingly where data allows.

The BSGM is neither without error nor a replacement for urban feature/built-settlement extractions methods. Given that the BSGM modelling framework is interpolative, its modelled BS extents are limited by the accuracy, the spatial and temporal resolution of its inputs including the RS-based observed BS extents, the time specific subnational population data, and the spatially-explicit population distribution dataset. For example, the poorer model performance from 2001 through 2003 (Table 4 and Fig. 5) is likely due to the fact the ESA RS-based BS extents were delineated at 30 arc sec resolution, due to the MERIS and PROBA V imagery not being available, rather than the 10 arc sec resolution for years from 2004 through 2015 (UCL Geomatics, 2017). With regards to the total disagreement of the BSGM-based BS extents to the ESA RS-based BS extents (Fig. 5), the relatively low contribution of allocation disagreement prior to circa 2010 and corresponding increase in contribution post-2010 is possibly due to the switch from using coarser DMSP-based LAN data to VIIRS-based LAN data at the 2012 time point.

The BSGM modelling framework is also limited by conceptual and mathematical assumptions. We are assuming a certain relationship between relative BS population and BS population density changes and drive demand for temporally coincident BS growth. Furthermore, we assume that BS population grows logistically with a time varying capacity that is temporally coincident and that BS population density follows a natural cubic spline across all observed points. This is further predicated upon the assumption that the BS growth is strongly correlated by changes in population and or population density and the resulting demand is instantaneously filled as opposed to being delayed temporally. While there is support for population change being an empirical and theoretical driver of BS growth (Angel et al., 2011; Dyson, 2011; Linard et al., 2013; Seto et al., 2011, Seto et al., 2012), there is also evidence for considering other drivers, not used here because of their unavailability at subnational levels globally through time, such as Gross Domestic Product and arable land per capita (Angel et al., 2011; Seto et al., 2011). Furthermore, there are other “intangibles” such as local, regional, and national land use or development policies, which almost certainly shape the BS growth, but are typically not available in an accessible format or not available at all. Furthermore, the BSGM modelling framework is relying on temporally (Doxsey-Whitfield et al., 2015) and spatially (Stevens et al., 2015) modelled subnational population data that are used as inputs to estimate the BS population at each point in time. However, regardless of the modelling approach used to spatially disaggregate the population from the unit to the pixel-level, since the BSGM modelling framework allocates transitions based upon relative changes in BS population, the errors associated with the spatial redistribution of the population should not affect prediction timings, as long as biases are consistent over times. As with any “model outputs built upon model outputs,” users of such datasets must be cautious of accumulated errors.

When considering area-based metrics, the Modifiable Areal Unit Problem (MAUP) (Openshaw, 1984) must be considered. Indeed, the total number of pixels in each unit is typically larger in the less settled units, resulting in less variation of aggregated metric values referring to those. With dasymetric redistribution methods, the size and spatial arrangement of the source units, can also affect the quality of the disaggregation with the larger relative differences between source unit and target unit sizes introducing more (Mennis, 2003; Mennis & Hultgren, 2006). This, in part, likely led to the results in Fig. 6. The MAUP could also explain the framework's late prediction regarding infill growth (Fig. 7), with the unit-averaging of the BS population density potentially obscuring the underlying sub-unit variation (Openshaw, 1984) in BS population density that could be more likely driving pixel-level non-BS-to-BS transitions. Other reasons for disagreements in Fig. 7 could be due to less detectable light changes associated with non-BS-to-BS transition due to light blooming. However, annual BSGM-based BS extents can be aggregated across years to decrease uncertainty of the interpolated extents, as the growth of BS extents is an incremental process, with future outcome dependent upon previous growth.

Unfortunately, for both the use of logistic curves to interpolate between estimates of BS population count data and the use of cubic splines to interpolate between estimates of BS population density data, independent data does not exist to evaluate the error or uncertainty of the interpolated values. This aside, we also cannot calculate uncertainty of these curves because they are non-parametric growth curves or simple fitted splines that are not conducting any statistical inferences. However, we are not actually using the interpolated values of BS population and BS population density as the predicted outcome of interest, but rather to derive estimated counts of non-BS to BS pixel transitions that are then used as relative weights for the spatial disaggregation of the actual RS-based observed transition counts across time (Eqs. (1), (2), (3), (4), (5), (6), (7)). Finally, it is important to highlight how this dasymetric disaggregation by weights precludes the propagation of any uncertainties calculated before the disaggregation step (as a well-known characteristic of dasymetric methods), limiting us to only measuring absolute error of the final transitions as we have done here.

## 5 Conclusions

The 2030 Agenda for Sustainable Development and its SDGs, have reinforced the importance of data to being able to account for “all people everywhere (United Nations, 2016). Differences in the dynamic spatial distributions of hazards (Carrão, Naumann, & Barbosa, 2016; Oliveira, Oehler, San-Miguel-Ayanz, Camia, & Pereira, 2012), the spatial variation of the effects of climate change (Ericson, Vorosmarty, Dingman, Ward, & Meybeck, 2006; Hanjra & Qureshi, 2010; Stephenson et al., 2010), spatially allocating services to ensure sufficient coverage (Eckert & Kohler, 2014; Sverdlik, 2011), and targeting interventions and planning (Linard, Alegana, Noor, Snow, & Tatem, 2010; Utazi et al., 2018) based upon local context with limited resources requires higher temporal resolution in the mapping of BS and mapping of populations, both large and small (United Nations, 2016). Here we described a flexible modelling framework for globally modelling BS extents between RS-based observed time points, with 39 of 48 validated BSGM predicted BS extents having over 80% agreement with ESA RS-based observed extents and 25 of those years having over 90% agreement (Table 4). This framework is scalable globally, but also allows for sub-national variation in transition probability, population changes, and local relative LAN changes to drive the overall study area model.

As global urban feature/built-settlement extent datasets such as ESA CCI, MAUPP, GHSL, GUF and others continue to improve both in terms of spatial accuracy and spatial and temporal resolutions, modelling frameworks such as the BSGM will likely still be useful due to imagery/extraction issues and the need to smooth or fill-in time-series of urban feature/built-settlement datasets (ESA CCI, 2017; Esch et al., 2013; Forget, Linard, & Gilbert, 2018; Pesaresi et al., 2016). By the time annual urban feature/built-settlement extractions from currently available imagery will become an economically viable means of filling gaps, the current demand for annual datasets, eventually becoming the standard, will be replaced by grown a demand for quarterly and monthly datasets. This is not to say that interpolative models and feature-extraction algorithms are oppositional, but rather that they are complementary. Should the time come where high-resolution global annual urban feature/built-settlement datasets become the norm, this would offer a wealth of information from which to improve the assumptions the BSGM currently makes.

As informative as global RS-based urban feature/built-settlement datasets are, imagery will never see into the future and we plan on extending the BSGM modelling framework to allow for short-term projection of the growth of BS extents. We found that the primary predictors of growth BS extents were related to connectivity, i.e. road networks, and local, i.e. ~0.5–1.5 km, settlement density (Fig. 4) giving support to work in attempting to define “urban” based on contiguity, connectivity, and spatial density (Dijkstra & Poelman, 2014; Esch et al., 2014; Pesaresi & Freire, 2016). Still mostly unknown is how the BSGM modelling framework would perform for smaller settlements, not captured by the coarser datasets such as the ESA CCI land cover, and we are looking to test this with forthcoming feature data sets with resolutions below 3 arc sec. Further sensitivity testing of the framework to noisy or biased inputs, e.g. BS datasets in arid biomes, is also planned. Lastly, we plan to validate the utility of these dataset in an applied manner by comparing the effects of including the BSGM-based BS extents in annual population distribution modelling. Finally, the BSGM modelling framework can be adapted to run at other scales, both spatially and temporally, either by modifying the provided code (See Supplemental Materials) or, in many cases, simply by modifying the input data. Annual global interpolated datasets from 2000 to 2014 based on GHSL/ESA/GUF input datasets, produced with an early version of this model and a reduced set of covariates, is freely available on the WorldPop website (worldpop.org) with the model code and results datasets used here provided in the Supplemental Material repository at.

## Author contributions

JJN, AS, JES, and AJT designed the research. FRS, AEG, CL and JJN contributed to previous model concepts that resulted in the presented model realization. DC and AC contributed significant knowledge transfer on bootstrapping and growth curves. JJN carried out analyses and research. JJN, MB, and TE provided data and or carried out data pre-processing. JJN wrote the modelling script with MB providing the code framework for the larger scale data production. JJN wrote the manuscript with contributions and edits from all other authors.

## Appendix A Supplementary data

Supplementary material Image 1
