# Supplementary material for: Annually modelling built-settlements between remotely-sensed observations using relative changes in subnational populations and lights at night
Source: Comput Environ Urban Syst. 2020 Mar;80:101444. doi: 10.1016/j.compenvurbsys.2019.101444 (PMC7043396; doi:10.1016/j.compenvurbsys.2019.101444)
Supplement: Supplementary file 1 — Supplementary material [file mmc1.docx]

**Appendices**

***Section A1 – Covariates Used in Population Map Creation and Their Sources***

**Table A1.** Covariates utilized in the production of the population maps that were used as inputs into the built-settlement growth model

| **Covariate** | **Time Point(s)^a^** | **Original Source** | **Source Resolution** |
| --- | --- | --- | --- |
| DTE Cultivated landcover | 2000, 2005,2010, 2015 | ESA CCI Landcover (ESA CCI, 2017) classes 10-30 | 10 arc seconds |
| DTE Woody, Herbaceous, Shrub landcover | 2000, 2005,2010, 2015 | ESA CCI Landcover (ESA CCI, 2017) classes 40-120 | 10 arc seconds |
| DTE Grassland landcover | 2000, 2005,2010, 2015 | ESA CCI Landcover (ESA CCI, 2017) class 130 | 10 arc seconds |
| DTE Lichens and Mosses landcover | 2000, 2005,2010, 2015 | ESA CCI Landcover (ESA CCI, 2017) class 140 | 10 arc seconds |
| DTE Sparse Vegetation landcover | 2000, 2005,2010, 2015 | ESA CCI Landcover (ESA CCI, 2017) classes 150-153 | 10 arc seconds |
| DTE Aquatic Vegetation landcover | 2000, 2005,2010, 2015 | ESA CCI Landcover (ESA CCI, 2017) classes 160 - 180 | 10 arc seconds |
| DTE Bare Areas | 2000, 2005,2010, 2015 | ESA CCI Landcover (ESA CCI, 2017) class 200 | 10 arc seconds |
| DTE Built-settlement | 2000, 2005,2010, 2015 | ESA CCI Landcover (ESA CCI, 2017) class 190 |  |
| Distance to Inland Water Bodies | 2015, assumed invariant | MERIS-based water bodies (Lamarche et al., 2017) | 5 arc seconds |
| Distance to Roads | Downloaded 2017, assumed invariant as temporally specific road data unavailable | OpenStreetMap (OpenStreetMap Contributers, 2017) | Vector |
| Distance to Rivers | Downloaded 2017, assumed invariant | OpenStreetMap (OpenStreetMap Contributers, 2017) | Vector |
| Distance to Coastline | Based upon boundaries of GPWv4, assumed invariant | CIESIN GPWv4 (Doxsey-Whitfield et al., 2015) | Vector |
| Slope | 2000, assumed invariant | World Wildlife Fund Void-filled Hydrosheds (Lehner, Verdin, & Jarvis, 2008) | 3 arc seconds |
| Elevation | 2000, assumed invariant | World Wildlife Fund Void-filled Hydrosheds (Lehner et al., 2008) | 3 arc seconds |
| DTE: Distance To nearest Edge  a Note, for any covariate derived from land cover or built-settlement, only one year-specific covariate was used corresponding to the desired population surface (e.g., for a 2000 population surface only covariates corresponding to 2000, or those assumed temporally invariant, were used as covariates). | | | |

For every population year modelled, we included the distance to nearest BS edge for the year 2000, as population relates to older parts of a BS agglomeration differently from younger ones (Gaughan et al., 2016). For example, if we were to model the population map of 2010 we would include the distance to nearest observed BS edge for 2010 as one of the predictive covariates as well as the distance to nearest BS edge corresponding to the observed 2000 BS extents. This was done to avoid centres of agglomerations being assigned artificially low population densities relative to the preceding modelled time point (Gaughan et al., 2016).

***Section A2 – Full Process Diagram and Additional Rationale***


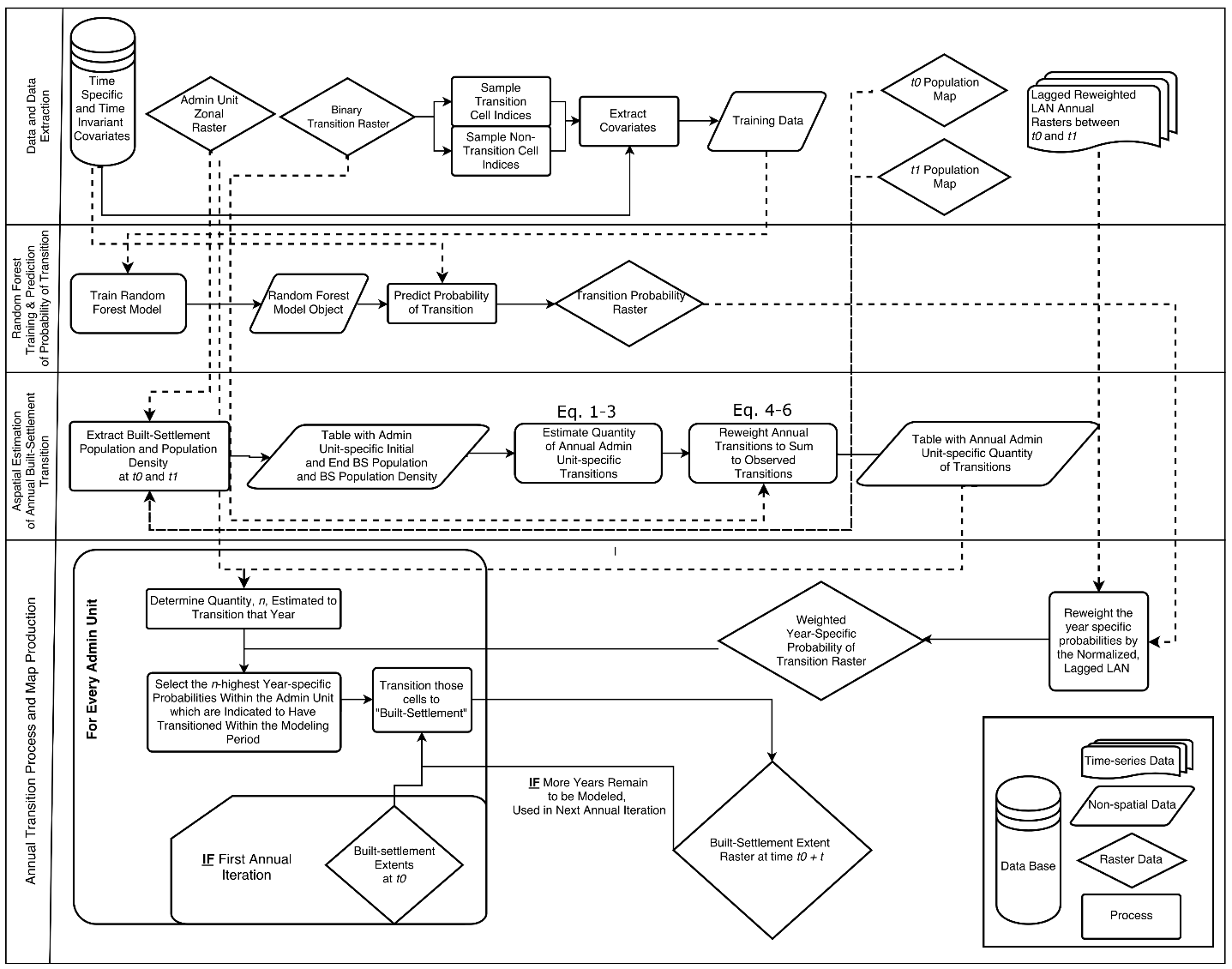
**Figure A1.** Overview of the generalized modelling process for a case of only two observed timepoints, *t0* and *t1*, with references to utilized equations.

The choice for equal sampling of each stratum was determined by testing different relative proportions and samples sizes until finding the most consistent and best model results, balancing performance and efficiency.

Logistic growth curves are widely used and accepted for modelling populations within demography, ecology, and urban modelling (Austin & Brewer, 1971; Cohen, 1995; Ledent, 1982; Smith, 1997; Wilson, 1976). Batty (2009) summarized, “Constrained population growth reflecting both exponential change and capacity which, in turn, reflect densities and congestion are simulated using various kinds of logistic growth.” because, as Sibly and Hone (Sibly, Barker, Denham, Hone, & Pagel, 2005) note, “While environmental stressors have negative effects on population growth rate, the same is true of population density, the case of negative linear effects corresponding to the well-known logistic equation.” as put forth first by Verhulst (1838). Furthermore, Ledent (1982) showed that urbanization, the process of *population* becoming urban, across time can be adequately summarized by “S-shaped curves”, specifically the functional logistic form. We followed this same underlying conceptual logic using a logistic curve with a dynamic limiting factor (Equation 1), i.e. the total population of an area is the theoretical limit of the temporally coincident BS population count.

For each unit, we interpolated the corresponding unit-average BS population densities, referring to the years *t* in *T*, across all unobserved years *t_k_*. Because there is a lack of literature and data on the actual or theoretical limits of human population density, we selected natural cubic splines to interpolate each unit’s BS population density while avoiding the sharp rates of change, that would be seen with piece-wise linear interpolation, or large oscillations seen with higher order polynomials (i.e. Runge’s phenomenon). Cubic splines have a long history in demographic interpolation, including interpolation of rates associated with urbanization processes (Ledent, 1982; McNeil, Trussell, & Turner, 1977). Here we are assuming that the trend of population density change of a given subnational unit is smooth and continuous across time, because short of some drastic (and unlikely and largely unaccountable “population shocks” such as wars or natural catastrophes) event, at the annual time scale we would not expect to see “cliffs” of population density change.

***Section A3 - Handling of Negative or “Decay” Transition Cases***

The process resulting from Equations 1-3 can produce "negative" predicted growth, hereafter decay, in any given year, and that the input built-settlement extent data assumes that once an area has transitioned to built-settlement it remains built-settlement. To account for this, we used the input data to limit the model to show "stagnation," i.e. no growth, or growth. We managed three case types of decay according to the information presented in Table A3. Case I (A and B) included situations where the observed transitions were greater than zero and some or all estimated transitions were negative. Case IIs included situations where the observed extent transitions were zero and the estimated transitions were negative. For full details, read comments in model code.

**Table A2.** Case types of predicted built-settlement decay and how they were handled in the model.

| Case Type | Sub type | Description | Origin | Implication | Handling |
| --- | --- | --- | --- | --- | --- |
| I | A | All predicted years < 0; observations > 0 | Built population decreases, built settlement increases because of differences in imagery and sensitivity of original datasets or because relationship between population and built settlement area are inverse of what would be expected. | Lacking any other information, we will assume that the greatest built settlement changes occurred circa the biggest population magnitude changes | The reweighting scheme makes all the weights positive by virtue of all individual differences being negative; no special action is necessary. |
|  | B | Some predicted years < 0; observations > 0 | Comes about from population decreasing for a year while outpacing the predicted decrease in built settlement population density | Built settlement growth during this period is unlikely compared to other years in the total transition period. | Set the predicted difference for that year, and therefore its weighted difference, to zero. |
| II | --- | Some, but not all, predictions < 0; Observations = 0 | Relationships between population and built settlement counts are not straightforward and not necessarily stationary through time and or space. Further, inaccuracies exist in the original built settlement data and the popA6ulation estimates. Any of these errors, in conjunction with model assumptions, could combine to result in this. | Continue with the base assumption that the input built-settlement data is the best we have in knowing if any transition occurred. | Set all predicted differences to zero in order to match the observed changes |

***Section A4 – Stochastic Process for Obtaining Agreement After Rounding of Predicted Transitions***

After the negative values were handled (See Supplemental Material, section A3) and the observed transitions were dasymetrically redistributed, sometimes there remained discrepancies, i.e. over or under estimations, between the sum of predicted transitions and the observed changes due to rounding during the weighting procedure. Here, we obtained agreement between the predicted and observed transitions by way of a stochastic process where, as long as the predicted number of transitions of a given administrative unit did not equal the corresponding observed transitions, we randomly selected a time point within the modelling period. We then added or subtracted, whichever was appropriate, one transition to the total predicted transitions for that year, $\hat{BSCNT_{ti}}$. This "salting" continued until agreement between the predicted and observed counts for a given admin unit was obtained. When we performed subtraction to correct for overestimation, we did not subtract from years that we already predicted to have no transitions.

***Section A5 – Additional Results Based Upon ESA Input to BSGM***

Overall, at the subnational unit level, we found results similar to the pixel-level results, including poor performance in absolute terms between 2001 to 2003, but some units were obviously performing worse than others as compared to the naive model. Plotting the ESA-informed model distributions of unit-level F1 scores by study area and year against the corresponding naive model performance, we show that the BSGM generally performs better in the majority of subnational units from which the transitions were disaggregated from (Figure A3). At worst, e.g. Vietnam 2002, approximately half of the units were still performing better than the naive model (Figure A3). For quantity disagreement (Figure A4) and allocation disagreement (Figure A5), results similar to pixel level results were found.


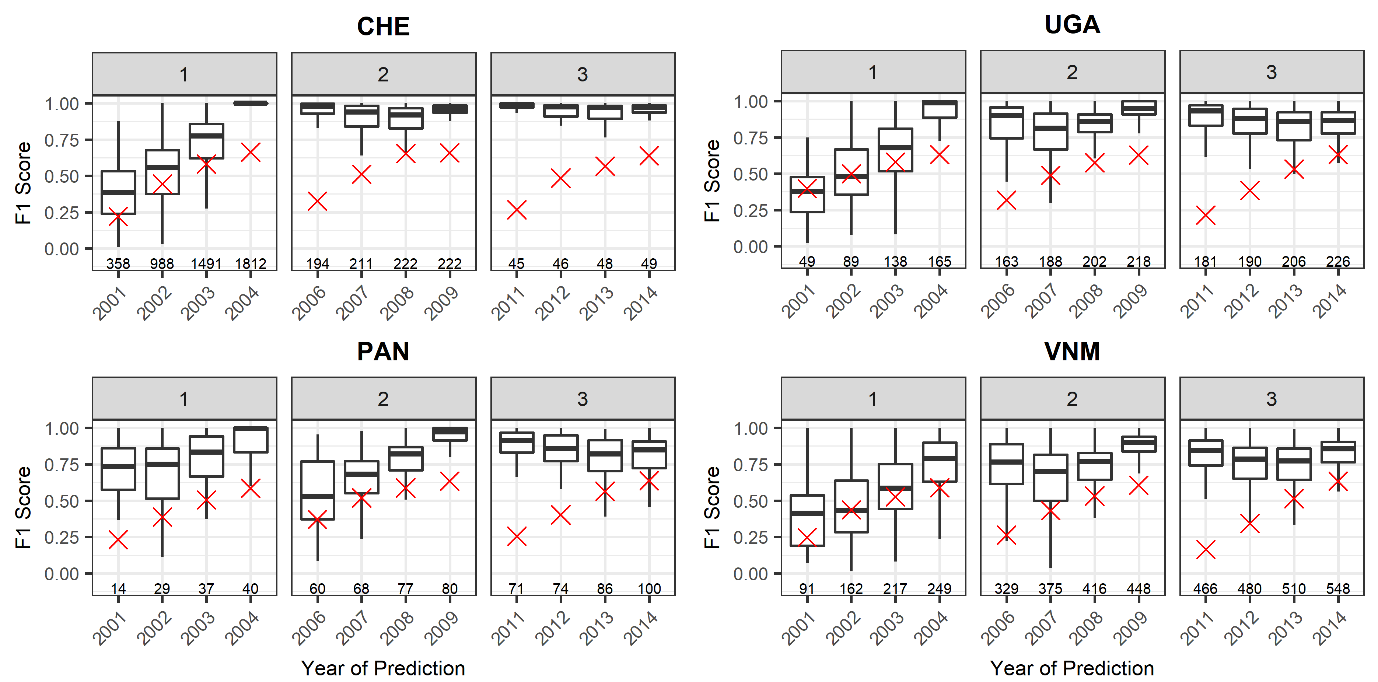
**Figure A3.** Unit level F_1_ score box plots, by dasymetric period, of Switzerland (CHE), Panama (PAN), Uganda (UGA), and Vietnam (VNM) ESA informed models as compared to a naive model, given by a red “x”. Number of units exhibiting any transitions for each period and a defined metric value is given above the x-axis.


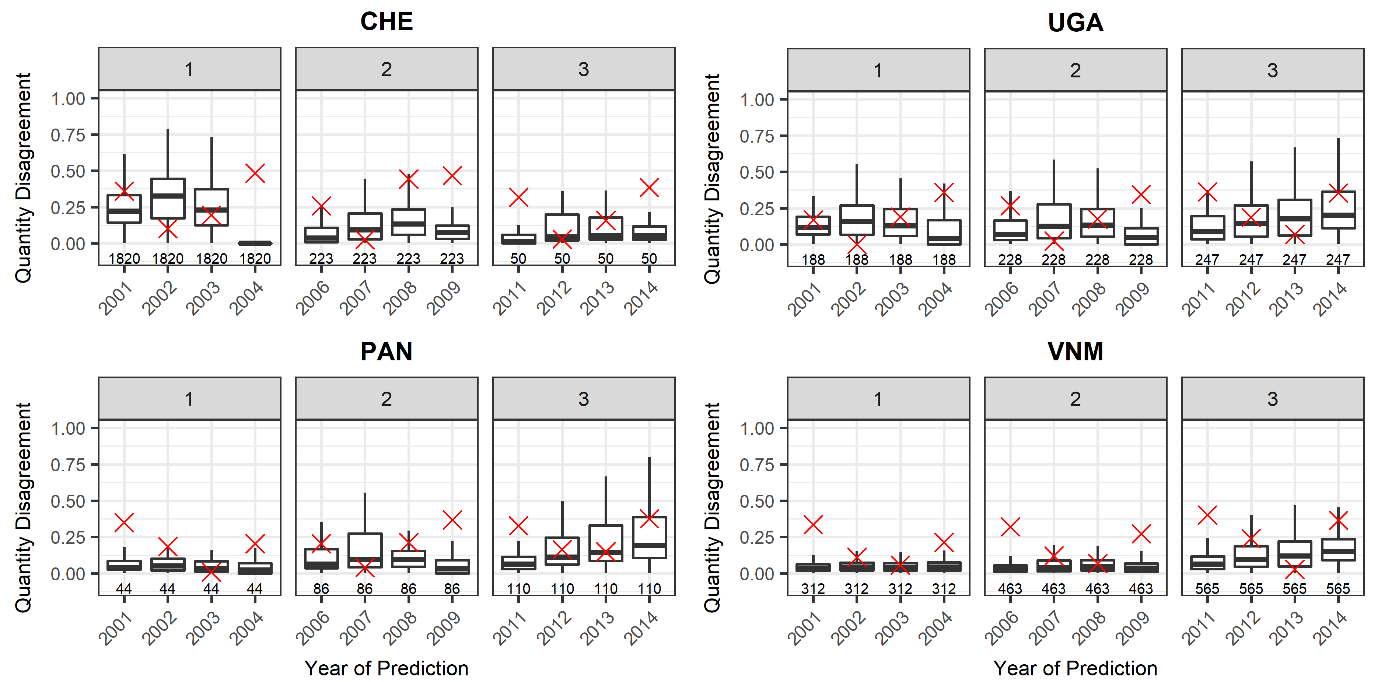


**Figure A4.** Unit level quantity disagreement box plots, by dasymetric period, of Switzerland (CHE), Panama (PAN), Uganda (UGA), and Vietnam (VNM) ESA informed models as compared to a naive model, given by a red “x”. Number of units exhibiting any transitions for each period and a defined metric value is given above the x-axis.


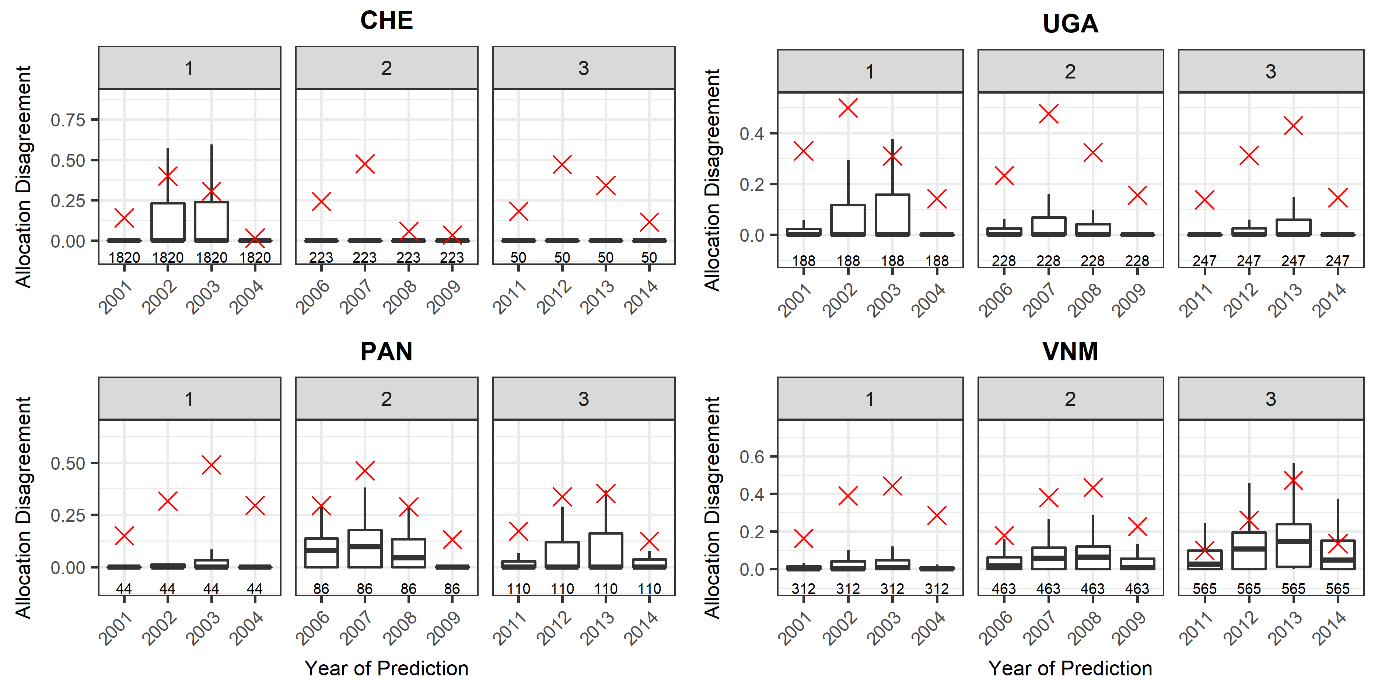
**Figure A5.** Unit level allocation disagreement box plots, by dasymetric period, of Switzerland (CHE), Panama (PAN), Uganda (UGA), and Vietnam (VNM) ESA informed models as compared to a naive model, given by a red “x”. Number of units exhibiting any transitions for each period and a defined metric value is given above the x-axis.

Plotting the unit-level metrics for all models as choropleth maps (see Supplementary Material for select maps and shape files containing contingency data), shows that years of generally good performance, the units of lesser performance are those that correspond to areas of less densely settled areas and the peripheries of established urban areas. Other years, such as Uganda 2001, performed poorly across many units with no apparent pattern.


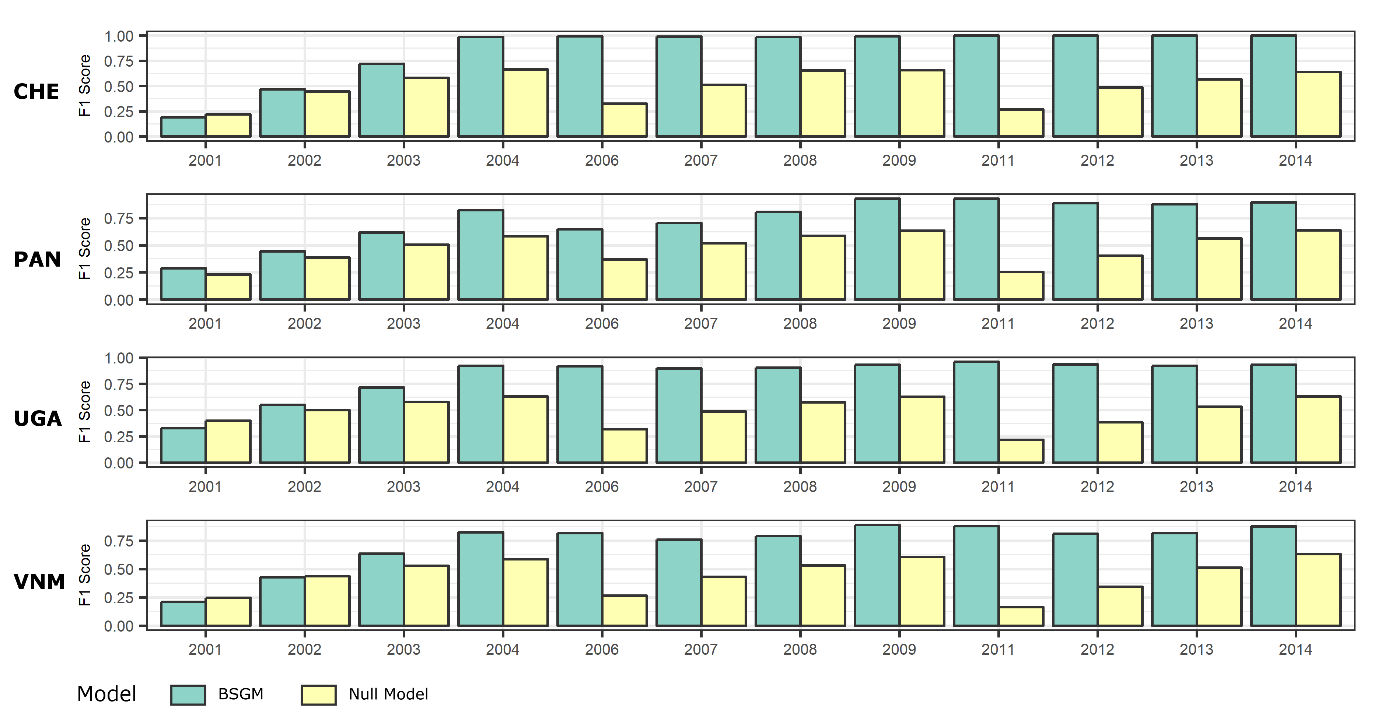
 Examining the year-specific study area F1 scores (Figure A6), we show that the BSGM modelling framework had low absolute performance and near naive model performance between 2001 and 2003 across all countries. After 2003, the F1 score notably, with values approaching 1.0 in some cases, and the BSGM modelling framework dramatically outperforms the naive model (Figure A6).

**Figure A6.** Pixel-level F_1_ score by year for the BSGM-based BS extents and BS extents produced using the naïve model for Switzerland (CHE), Panama (PAN), Uganda (UGA), and Vietnam (VNM) Full annual contingency data and metrics in supplementary material

***Section A6 – BSGM Results with GUF Evolution***

To demonstrate the flexibility of the modelling framework and its applicability to higher resolution, e.g. sub-100m, urban feature data, we also tested an alpha version, of the forthcoming World Settlement Footprint multi-temporal dataset, known as WSF Evolution (Esch, Bachofer, et al., 2018), hereafter WSF Evo. Starting in 2018 with a release of the WSF 2015 (equivalent of binary GUF, based on a joint analysis of multi-temporal Sentinel-1 and Landsat-8 data for the year 2015), the WSF-Evo product provides detailed information about the spatio-temporal development from 1985–2015 for each human settlement identified in the WSF-2015. The corresponding analysis is based on a processing of multitemporal collections derived from the Landsat archive using an implementation of the TimeScan approach (Esch, Üreyen, et al., 2018) at the Google Earth Engine (Gorelick et al., 2017) to generate the baseline layer for the classification. This classification starts by using the WSF 2015 as training data for the identification of the built-up area in 2010, and the using the resulting WSF 2010 as training input for classifying the 2005 data, and so on. The WSF Evo. Data used here covers a 50km x 50km rectangular area centred over Ho Chi Minh City for the dates of 2000, 2010, and 2015. The data was resampled to 3 arc seconds using nearest neighbor resampling and derived covariates were calculated from this. For the purposes of modelling, we only utilized areas that completely covered the subnational units in the population data. Summaries of the study areas with regards to BS transitions as defined by WSF Evo. are given in Table A3.

**Table A3.**  Descriptive summary of the WSF Evolution dataset where areal units are pixels pixels (~100m) as that is the unit handled by the model which looks at relative areal changes as opposed to absolute areal changes.

| Dataset | Country | Period | Initial Non-Built Area (pixels) | Observed Transitions |
| --- | --- | --- | --- | --- |
| WSF Evo. | Vietnam ^a^ | 2000-2015 | 3,295,142 | 10.43 % |
| a Ho Chi Minh City and immediate surroundings | | | | |

The RF using WSF Evo. Data, out of all the models, has the largest area under its PRC curve, but the precision begins to decrease, albeit less sharply, at lower recall levels than the ESA models (Figure A7).

**
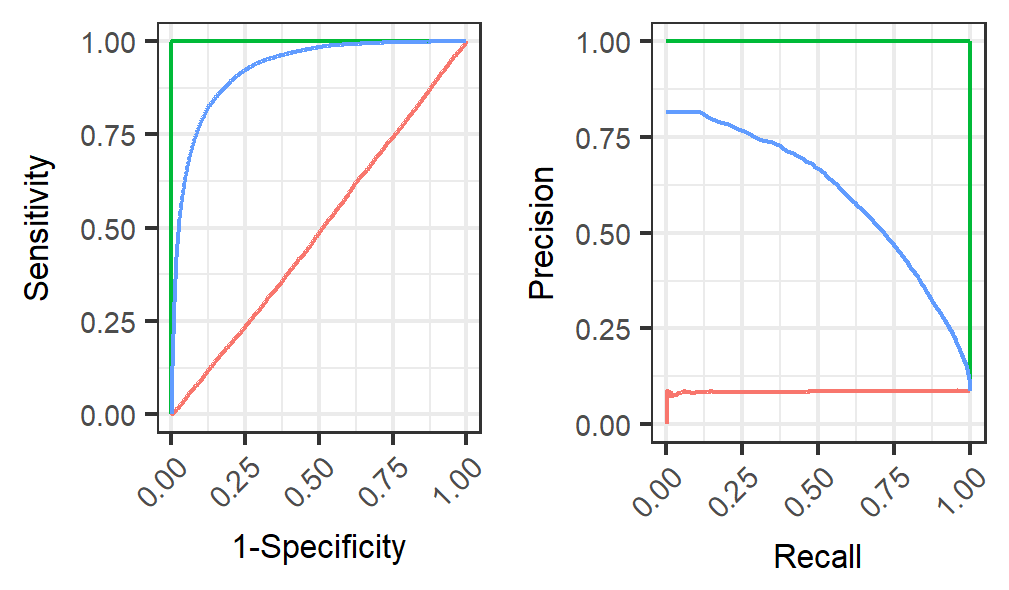
**

**Figure A7** Receiver Operator Curve (left plots) and Precision Recall Curves (right plots) with the RF model performance, blue lines, against a random model, red lines, and a perfect model, green lines, for each modelled country and input dataset

Covariate importances for the WSF Evo. (listed as GUF or GUF+ in Figure A8) model were comparable to that seen in the ESA models (Figure A8).


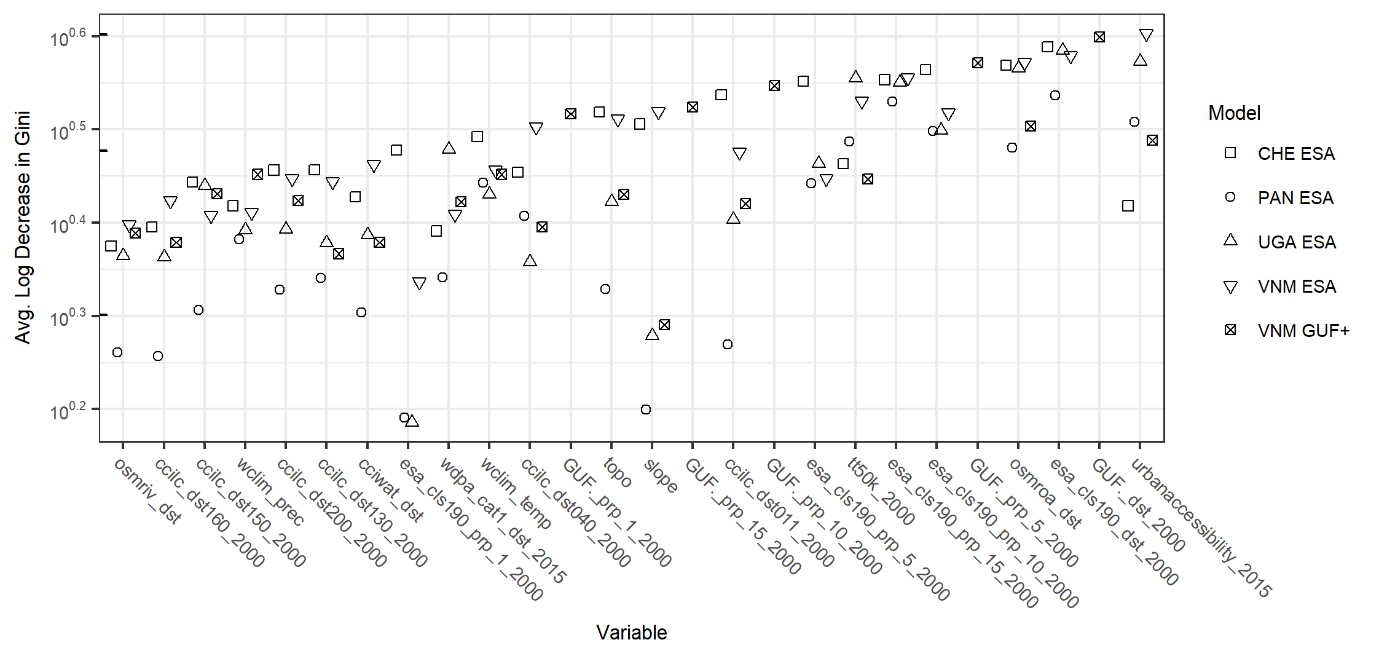
**Figure A8.** Random forest covariate importance as measured by the average log decrease in the Gini impurity when the covariate is used as the splitting criteria at nodes; higher values indicate better performance of covariate. Model for Swizerland (CHE) ESA, Panama (PAN) ESA, Uganda (UGA) ESA, Vietnam (VNM) ESA, and Vietnam WSF Evo. (GUF+) are shown. Refer to Table 1 for covariate names.

Overall, at the pixel level WSF Evo. Model performed much poorer than the ESA models with an overall accuracy of 0.518(Table A4).

**Table A4.** Proportion of transition pixels predicted correctly by the BSGM by year. Note that 1 – the proportion correct is equal to the overall disagreement, i.e. the sum of the quantity and allocation disagreement.

| Model | 2001 | 2002 | 2003 | 2004 | 2006 | 2007 | 2008 | 2009 | 2010 | 2011 | 2012 | 2013 | 2014 |
| --- | --- | --- | --- | --- | --- | --- | --- | --- | --- | --- | --- | --- | --- |
| CHE ESA | 0.718 | 0.573 | 0.628 | 0.975 | 0.987 | 0.979 | 0.975 | 0.983 | --- | 0.999 | 0.998 | 0.997 | 0.997 |
| PAN ESA | 0.952 | 0.935 | 0.934 | 0.960 | 0.806 | 0.771 | 0.816 | 0.920 | --- | 0.905 | 0.838 | 0.801 | 0.818 |
| UGA ESA | 0.814 | 0.787 | 0.803 | 0.929 | 0.912 | 0.877 | 0.877 | 0.909 | --- | 0.940 | 0.893 | 0.865 | 0.878 |
| VNM ESA | 0.942 | 0.918 | 0.923 | 0.951 | 0.923 | 0.872 | 0.866 | 0.916 | --- | 0.879 | 0.777 | 0.738 | 0.790 |
| VNM WSF Evo. | --- | --- | --- | --- | --- | --- | --- | --- | 0.518 | --- | --- | --- | --- |

For the single compared year modelled using WSF Evo. data, the F1 score performance is low both in absolute terms, approximately 0.33, and in relative terms, having a score approximately 0.05 higher than the null model (Figure A9).


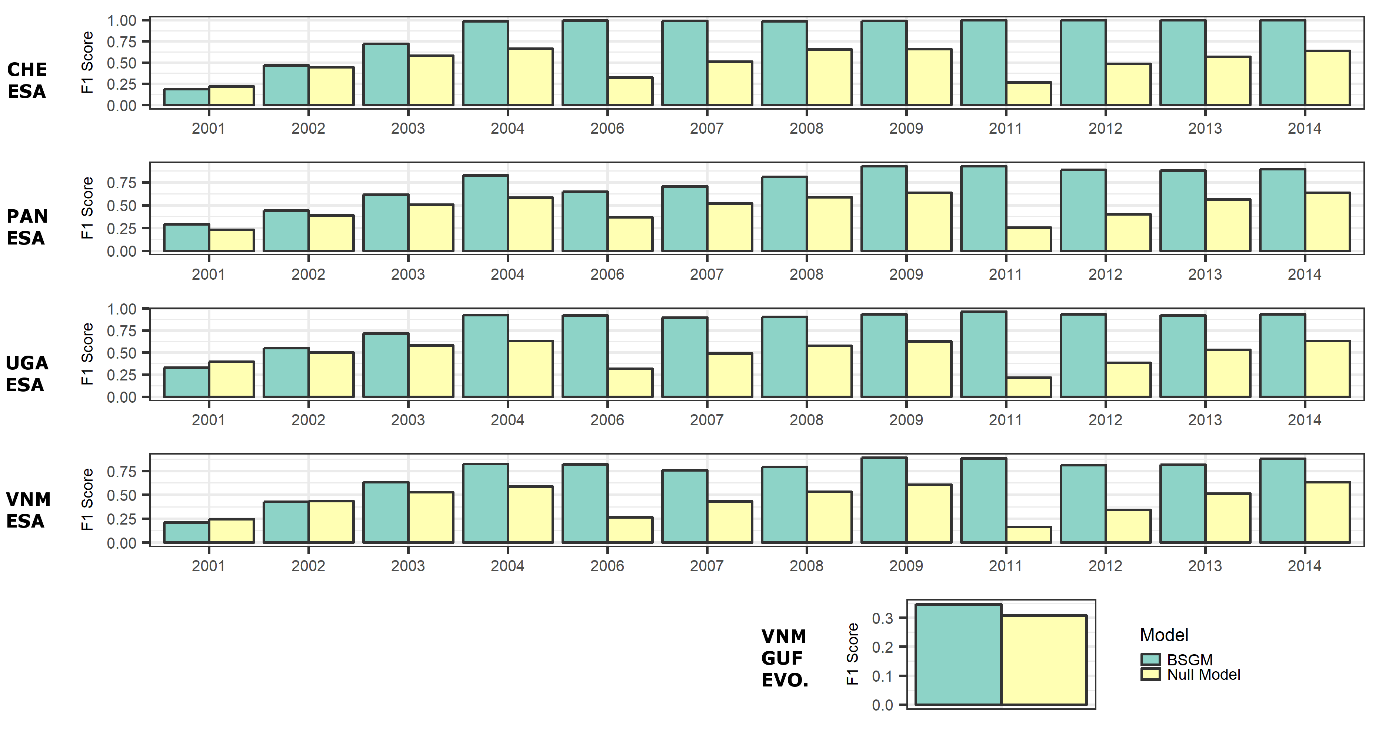


**VNM
WSF
EVO**

**Figure A9.** Pixel-level F_1_ score by year for Switzerland (CHE), Panama (PAN), Uganda (UGA), and Vietnam (VNM) as compared to a null model.

For the year modelled using WSF Evo., the total disagreement due to the BSGM is only slightly less than the null model primarily due to less allocation error (Figure A10).


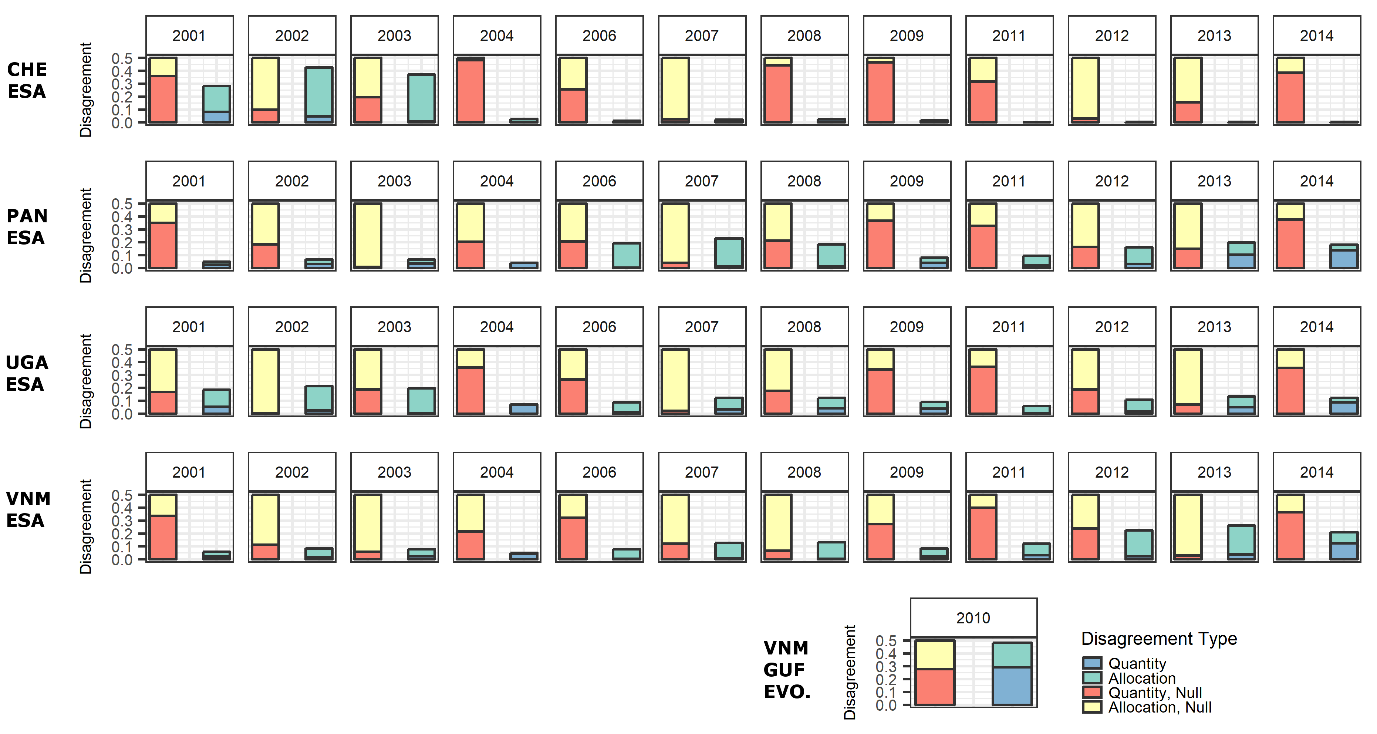


**VNM
WSF
EVO**

**Figure A10.** Pixel-level quantity and allocation disagreement of BSGM and null models for Switzerland (CHE), Panama (PAN), Uganda (UGA), and Vietnam (VNM) as compared to a null model, given in red. Full annual contingency data and metrics in supplementary material

For the WSF Evo. model, most of the units perform better than the null model in terms of F1 score and allocation disagreement (Figure A11). Conversely, many units have higher quantity disagreement than the null model, pointing to the underlying model performance issue being related to the population and population density interpolation as related to the assumed relationships with the input BS data rather than the RF and LAN allocation portion of the model.


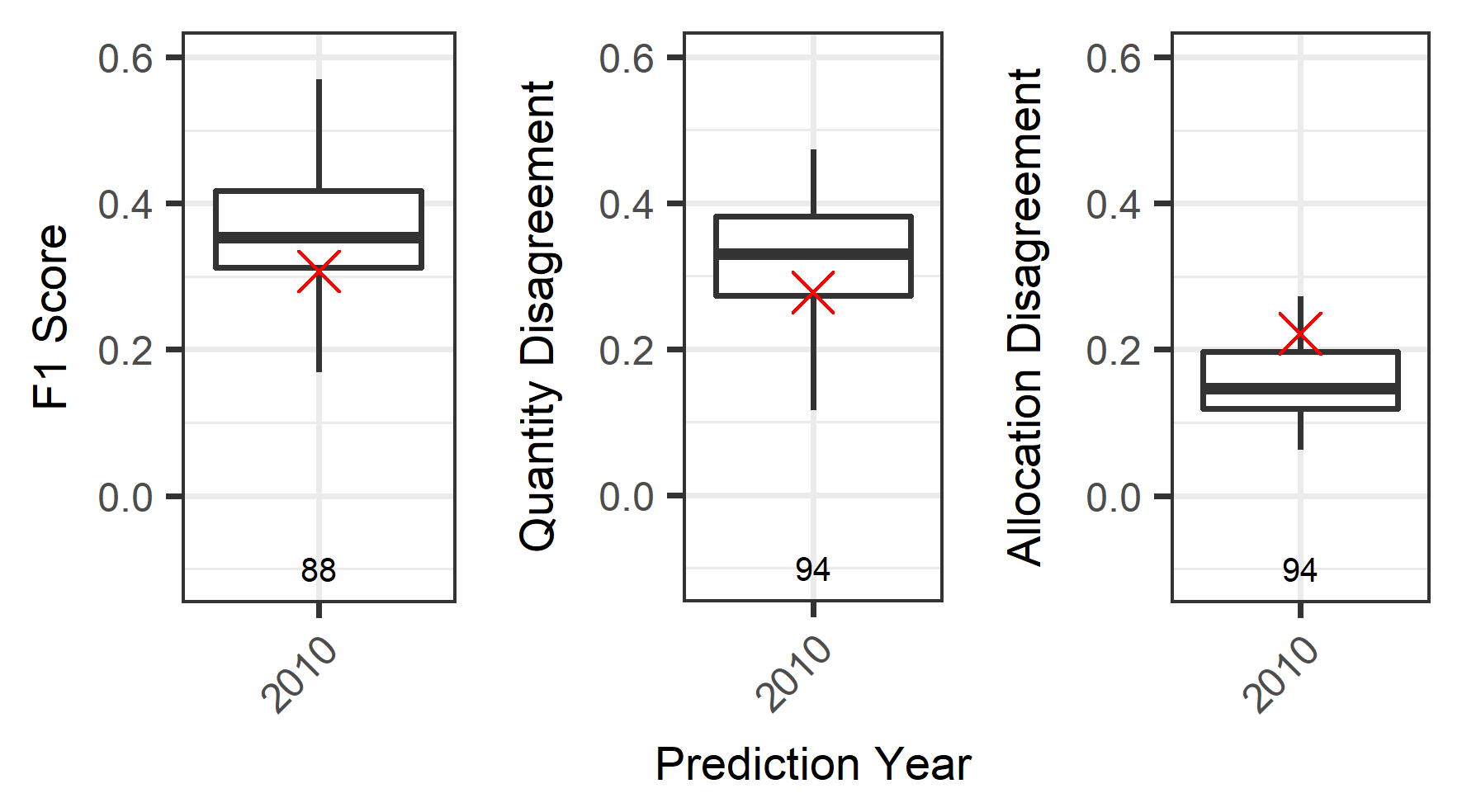


**Figure A11.** Using WSF Evo data, unit level distributions of F_1_ score, quantity disagreement, and allocation disagreement.

The poor performance of the BSGM with the WSF Evo. data (Figures A9-11; Table A4) was surprising considering the excellent performance with the ESA-informed models (Table A4; Figures A9-10). There could be many reasons for this, but we believe it originates because of a dissonance between the assumed population growth relationships and the input data in the form of one or the more of the following:

1. temporally differing biases in the modelled RF-informed population surface
2. less observed points to base the model upon
3. given the experimental nature of the WSF Evo. data, it is unvalidated and therefore how well it is capturing the BS extents at any given time point is largely unknown (but assumed to be better than the ESA class 190)
4. the WSF Evo data is capturing things perfectly, but the relationships the model can currently capture and describe are not sufficient to match the relationships between this spatial scale of data and the phenomenon.

More sensitivity analyses and validation testing is needed with finer scale input data across a larger time period of observations and a nested model, with varying behavior for small type BS agglomerations and large type agglomerations of BS, should be carried out.

***Section A7 –Training and Validation Sets of the Random Forest***

**Table A5.** Descriptive statistics of pre-existing prevalence of BS in the training and validation datasets used for measuring the performance of the RF models.

|  | Overall Prevalence at *t0 ^a^* | | | RF Training Set ^b^ | | | RF Validation Set ^c^ | | |
| --- | --- | --- | --- | --- | --- | --- | --- | --- | --- |
| Country | 0 Pixels | 1 Pixels | Prevalence of 1 | n | Pre-existing BS pixels | Pre-existing Prevalence | N | Pre-existing BS pixels | Pre-existing Prevalence |
| Panama | 8,901,004 | 23,805 | 0.27 % | 27,482 | 43 | 0.16% | 100,000 | 269 | 0.27% |
| Switzerland | 6,816,510 | 193,939 | 2.77 % | 100,000 | 1,464 | 0.2% | 100,000 | 2,847 | 2.85% |
| Uganda | 2,8231,555 | 26,831 | 0.10 % | 67,600 | 30 | 1.5% | 100,000 | 99 | 0.10% |
| Vietnam | 40,108,425 | 146,260 | 0.36 % | 100,000 | 199 | 0.04% | 100,000 | 341 | 0.34% |
| a 0 indicates no detected presence of built-settlement and 1 indicates detected presence of built-settlement  b Pre-existing means that built-settlement was already in the sampled pixel, at time *t0,* selected for the random forest training set and therefore could never transition  c Pre-existing means that built-settlement was already in the pixel, at time *t0,* sampled for the random forest validation set and therefore could never transition | | | | | | | | | |

***Section A8 – Modelling Times by Country***

Given the 20 covariates detailed in the paper and predicting with four observed points the following were the computational times for the model as run on a local computer with 32GB RAM and an 8 core i7-6700 3.4GHz processor.

**Table A6.** Descriptions of the number of units (pixels) in given sample countries and the computational efficiency of the modelling runs.

| Country | Total Pixels in Extent ^a^ | Total NonNA Pixels | Average Time per 100k non-NA pixels (secs)^b^ | Total Time HH:MM:SS (secs) |
| --- | --- | --- | --- | --- |
| Panama | 20,700,555 | 8,924,809 | 71.6 | 01:46:27 (6,387) |
| Switzerland | 13,056,459 | 7,010,449 | 13.9 | 01:05:30 (3,930) |
| Uganda | 44,666,840 | 28,258,386 | 18.3 | 01:26:23 (5,183) |
| Vietnam | 156,409,044 | 40,254,685 | 16.0 | 01:47:07 (6,427) |
| a Note: some of this pixels contain no data based upon the difference between the boundaries of the country and the rectangular extent of the raster  b The Panama times is much larger than other runs due to an unoptimized parameter that determines the number of blocks to divide the rasters into for the parallel prediction using the random forest. This parameter value has to be adjusted for a variety of considerations (total pixels, number of covariates, amount of no data values in raster extent, number of resulting blocks that can be skipped because they contain all no data values, etc.) with each model run. We did not optimize for Panama and Switzerland due to their small size and relative speed even when unadjusted. | | | | |

**References**

Austin, A. L., & Brewer, J. W. (1971). World Population Growth and Related Technical Problems. *Technological Forecasting and Social Changes*, *3*(1), 23–49.

Batty, M. (2009). Urban Modeling. In *International Encyclopedia of Human Geography* (pp. 51–58). Oxford, UK: Elsevier.

Cohen, J. E. (1995). Population Growth and Earth’s Human Carrying Capacity. *Science*, *269*(5222), 341–346.

Doxsey-Whitfield, E., MacManus, K., Adamo, S. B., Pistolesi, L., Squires, J., Borkovska, O., & Baptista, S. R. (2015). Taking advantage of the improved availability of census data: A first look at the Gridded Population of the World, Version 4. *Papers in Applied Geography*, *1*(3), 226–234. https://doi.org/10.1080/23754931.2015.1014272

ESA CCI. (2017). European Space Agency Climate Change Initiative Landcover. European Space Agency. Retrieved from http://maps.elie.ucl.ac.be/CCI/viewer/download.php

Esch, T., Bachofer, F., Heldens, W., Hirner, A., Marconcini, M., Palacios-Lopez, D., … Gorelick, N. (2018). Where We Live—A Summary of the Achievements and Planned Evolution of the Global Urban Footprint. *Remote Sensing*, *10*(6), 895. https://doi.org/10.3390/rs10060895

Esch, T., Üreyen, S., Zeidler, J., Metz–Marconcini, A., Hirner, A., Asamer, H., … Marconcini, M. (2018). Exploiting big earth data from space – first experiences with the timescan processing chain. *Big Earth Data*, *2*(1), 36–55. https://doi.org/10.1080/20964471.2018.1433790

Gaughan, A. E., Stevens, F. R., Huang, Z., Nieves, J. J., Sorichetta, A., Lai, S., … Tatem, A. J. (2016). Spatiotemporal patterns of population in mainland China, 1990 to 2010. *Scientific Data*, *3*. https://doi.org/10.1038/sdata.2016.5

Gorelick, N., Hancher, M., Dixon, M., Ilyushchenko, S., Thau, D., & Moore, R. (2017). Google earth engine: Planetary-scale geospatial analyss for everyone. *Remote Sensing of Environment*, *202*, 18–27.

Lamarche, C., Santoro, M., Bontemps, S., D’Andrimont, R., Radoux, J., Giustarini, L., … Arino, O. (2017). Compilation and Validation of SAR and Optical Data Products for a Complete and Global Map of Inland/Ocean Water Tailored to the Climate Modeling Community. *Remote Sensing*, *9*(36). https://doi.org/10.3390/rs9010036

Ledent, J. (1982). Rural-Urban Migration, Urbanization, and Economic Development. *Economic Development and Cultural Change*, *30*(3), 507–538. Retrieved from https://www.jstor.org/stable/3203205

Lehner, B., Verdin, K., & Jarvis, A. (2008). New Global Hydrography Derived from Spaceborne Elevation Data. *Eos, Transactions of the American Geophysical Union*, *89*(10), 93–94. https://doi.org/10.1029/2008EO100001

McNeil, D. R., Trussell, T. J., & Turner, J. C. (1977). Spline Interpolation of Demographic Data. *Demography*, *14*(2), 245–252. Retrieved from https://www.jstor.org/stable/2060581

OpenStreetMap Contributers. (2017). OpenStreetMap (OSM) Database. OSM. Retrieved from openstreetmap.org

Sibly, R. M., Barker, D., Denham, M. C., Hone, J., & Pagel, M. (2005). On the Regulation of Populations of Mammals, Birds, Fish, and Insects. *Science*, *309*(5734), 607–610. https://doi.org/10.1126/science.1110760

Smith, S. K. (1997). Further thoughts on simplicity and complexity in population projection models. *International Journal of Forecasting*, *13*, 557–565.

Verhulst, P.-F. (1838). Notice sur la loi que la population poursuit dans son accroissement. *Correspondance Mathe-Matique et Physique*, *10*, 113–121.

Wilson, A. G. (1976). Catastrophe Theory and Urban Modelling: An Application to Modal Choice. *Environment and Planning A*, *8*(3), 351–356. https://doi.org/https://doi.org/10.1068/a080351
